# Maternal Exercise and Nutrition Function as Interconnected Regulators of Embryonic Organogenesis and Fetal Development

**DOI:** 10.3390/nu18162671

**Published:** 2026-08-15

**Authors:** Anqi Wu, Yilan Guo, Chengyi Zhong, Peng Sun

**Affiliations:** 1 College of Physical Education and Health, East China Normal University, Shanghai 200241, China; 13505315810@163.com (A.W.); yilanguo1997@163.com (Y.G.); chengyiz2026@163.com (C.Z.); 2 The Key Laboratory of Adolescent Health Assessment and Exercise Intervention of the Ministry of Education, East China Normal University, Shanghai 200241, China

**Keywords:** maternal exercise, Developmental Origins of Health and Disease (DOHaD), fetal developmental programming, epigenetic reprogramming, maternal nutrition, diet–exercise interaction

## Abstract

The intrauterine environment plays a critical role in shaping lifelong health, forming the foundation of the Developmental Origins of Health and Disease (DOHaD) paradigm. Increasing evidence suggests that maternal lifestyle factors, particularly physical activity and nutrition, can actively influence embryonic development; however, how exercise and dietary signals interact to regulate developmental programming remains insufficiently understood. In this review, we summarize emerging evidence that maternal exercise and nutrition function as interconnected regulators of embryonic organogenesis through coordinated maternal–placental–fetal communication. Exercise-induced metabolic, endocrine, and immunological adaptations are highly dependent on maternal nutritional status, while dietary composition provides essential substrates and signaling molecules that modulate exercise-mediated benefits. Through regulation of placental function, exerkines, microbiota-derived metabolites, epigenetic modifications, and nutrient-sensing pathways, the integrated exercise–nutrition axis shapes the development of multiple embryonic organ systems and influences offspring health trajectories. We further discuss current challenges in defining optimal combinations of maternal exercise patterns and nutritional strategies, highlighting the need to move beyond isolated interventions toward precision lifestyle approaches during pregnancy. This perspective establishes maternal exercise and nutrition as synergistic modulators of developmental programming and provides a conceptual framework for understanding how combined lifestyle interventions may enhance offspring developmental resilience and reduce the risk of chronic disease across generations.

## 1. Introduction

The “Developmental Origins of Health and Disease” (DOHaD) theory posits that the early-life intrauterine microenvironment is a critical determinant of an individual’s lifelong health trajectory [1]. The embryonic and fetal periods are not only the critical windows for tissue and organ morphogenesis but also the sensitive phases for the establishment of metabolic homeostasis, the immune system, and neural networks. Epidemiological evidence indicates that an adverse intrauterine environment (e.g., maternal obesity, gestational diabetes mellitus, undernutrition, or overnutrition) can significantly increase the offspring’s risk of developing obesity, type 2 diabetes, cardiovascular diseases, and neurocognitive disorders in adulthood through developmental programming mechanisms, thereby creating an intergenerational vicious cycle of chronic non-communicable diseases. Therefore, identifying safe and effective prenatal interventions to disrupt this intergenerational transmission of diseases has become an urgent need in perinatology and public health [1,2].

For a long time, clinical attention has predominantly focused on maternal weight management and nutritional interventions during pregnancy, whereas the role of maternal exercise has often been limited to physical aspects such as controlling maternal weight gain or alleviating back pain [3,4]. With the intersection of exercise physiology and molecular biology, growing evidence suggests that maternal exercise is an active physiological stimulus capable of inducing systemic adaptations. It not only improves maternal cardiopulmonary function and metabolic status but also directly or indirectly reshapes the embryonic developmental microenvironment through complex molecular networks [5,6]. While the physical activity itself cannot traverse the placental barrier, exercise-triggered circulating bioactive molecules—including exerkines (e.g., apelin, irisin), metabolic intermediates (e.g., short-chain fatty acids, α-ketoglutarate), epigenetic mediators, and hormonal signals—can be transported across the maternal–fetal placental interface or originate from placental synthesis to regulate embryonic development. This distinction is critical: maternal exercise acts as a systemic physiological stimulus that generates a cascade of humoral factors, rather than directly “crossing” into the fetal compartment.

Recent breakthrough studies have provided novel perspectives for unraveling this mystery. All mechanistic statements in the following sections are accompanied by explicit species attribution to distinguish preclinical (rodent/mammalian animal) evidence from human clinical data. Epigenetic studies have confirmed that exercise-induced metabolic alterations (e.g., the accumulation of α-ketoglutarate) can modulate DNA methyltransferase activity, conferring long-lasting antioxidant and metabolic advantages to the offspring without altering the DNA sequence [7]. Furthermore, the maternal gut microbiota, emerging as a novel regulatory hub, has been proven to mediate the distal regulation of exercise on the fetal nervous and immune systems via its metabolites, such as short-chain fatty acids (SCFAs) [8,9]. Meanwhile, maternal nutritional status—such as protein intake, fatty acid profile, vitamin D, and dietary patterns—acts as a key determinant of the same developmental programming pathways. Emerging evidence suggests that nutrition and exercise do not operate independently but rather exert synergistic effects on the gut microbiota–metabolite axis and offspring skeletal DOHaD (human cohort and rodent mechanistic data), highlighting the need to integrate these two modifiable factors [10].

Based on these premises, this review aims to systematically summarize the latest research progress on how maternal exercise regulates embryonic development. By broadening the traditional maternal perspective, we deeply explore how exercise exerts specific protective effects on the multisystemic development of the offspring—including the nervous, cardiovascular, digestive, respiratory, and musculoskeletal systems—through three core mechanisms: optimizing placental endocrine function, reshaping the maternal–fetal immune microecology, and inducing embryonic cellular signaling pathways and epigenetic reprogramming. By delineating this intricate molecular regulatory network, this review expects to provide a theoretical basis for understanding the transgenerational health effects of exercise and to offer scientific references for formulating precision maternal exercise prescriptions based on molecular mechanisms in the future.

## 2. Methods

Literature consulted for this narrative review was retrieved from four electronic databases: PubMed, Web of Science, Scopus and Embase. Core combined keywords for retrieval: (“maternal exercise” OR “prenatal physical activity”) AND (“DOHaD” OR “embryonic developmental programming”) AND (“placenta” OR “epigenetics” OR “gut microbiota” OR “fetal organ development”). Eligible studies included human cohort trials, mammalian animal intervention experiments, cell molecular studies, and peer-reviewed systematic/narrative reviews published in English. Exclusion criteria: case reports, conference abstracts without full text, non-mammalian model research, articles only discussing postnatal exercise without gestational maternal intervention. No formal dual-reviewer stepwise screening procedure was conducted, and all references were screened and synthesized narratively by the authors.

### 2.1. Key Stages and Biological Characteristics of Embryonic Development

Human embryonic development begins with fertilization, followed by a series of highly ordered morphogenetic events, including cleavage, blastocyst formation, implantation, and gastrulation, which progressively establish the spatial blueprint of ontogeny. Gastrulation is the central node for the establishment of the locomotor system lineage: through the migration of primitive streak cells and epithelial–mesenchymal transition (EMT), the intact tri-laminar system of the ectoderm, mesoderm, and endoderm is successfully established. Specifically, the axial subdivision of the mesoderm directly determines the primordia distribution of future bones, myocardium, skeletal muscles, and connective tissues [11,12]. The paraxial mesoderm undergoes somitogenesis to form sclerotomes and myotomes, providing the structural prerequisites for vertebral development and the lineage specification of trunk musculature, whereas the lateral plate mesoderm supplies the cellular sources for limb bones and intrinsic muscles via limb bud-inducing signals. Following implantation, embryonic development enters a window of high sensitivity to environmental perturbations. During this phase, the fibroblast growth factor (FGF), bone morphogenetic protein (BMP), NODAL, and Wnt signaling pathways form a precisely cross-linked regulatory network, collectively maintaining the integrity, pluripotency, and lineage-specification balance of germ layer stem cells [13]. In the context of locomotor system development, subtle fluctuations in signal intensity during this stage can be manifested through downstream target genes (e.g., *Tbx6*, *Msgn1*, *Pax3*), thereby disrupting somite boundary formation, myogenic progenitor cell migration, and limb bud positioning. This consequently increases the risk of movement-related structural anomalies, such as congenital spinal deformities and limb dysplasia. Thus, the homeostasis of signaling during this critical window serves not only as a safeguard for embryonic morphogenesis but also as a molecular barrier for the normal pattern formation of locomotor organs [14,15,16]. As pregnancy progresses from the organogenetic period (embryonic period, weeks 3–8) smoothly to the fetal period (week 9 to birth), the core of embryonic development gradually shifts from structural morphogenesis to functional maturation and growth integration. During the fetal period, crucial events in the locomotor system—such as the establishment of central nerve-muscle junctions, the differentiation of muscle fiber types, the morphogenesis of joint cavities, and the expansion of primary ossification centers—undergo rapid development. Spontaneous embryonic limb movements begin to emerge; through activity-dependent synaptic pruning and calcium signal-mediated transcriptional regulation, these movements precisely sculpt the composition ratio of motor units and the afferent patterns of muscle spindles, forming the neural circuit foundation for postnatal postural control and motor output reserves [17]. As the upstream regulatory field for developmental plasticity, the delicate fine-tuning of metabolic, endocrine, and mechanical signals within the intrauterine microenvironment can exert long-term programming effects on fetal growth trajectories [18]. Clinical and basic research indicates that maternal nutritional status, glucose and lipid metabolism levels, placental blood supply, and the biomechanical environment of the amniotic fluid can regulate the insulin-like growth factor axis and the expression of myogenic regulatory factors through epigenetic modifications (such as DNA methylation and histone acetylation). Direct evidence from fetal cord blood and fetal circulation in both human and animal studies supports the trans-placental transmission of exercise-induced signals. In human pregnancies, cord blood analyses have revealed that maternal exercise is associated with altered fetal circulating metabolite profiles, including elevated levels of arginine and citrulline—substrates for nitric oxide synthesis—as well as modulated branched-chain amino acid concentrations. In rodent models, fetal serum from exercise-trained dams exhibits increased apelin concentrations and enhanced thyroid hormone (T3) signaling, which directly promote fetal myogenesis and metabolic programming. Furthermore, human cord blood epigenetic studies have demonstrated that maternal exercise induces genome-wide DNA methylation alterations in fetal hematopoietic cells, affecting genes involved in metabolic regulation and immune responses. These fetal circulation-derived data collectively confirm that exercise-induced molecular changes are not confined to the maternal compartment but actively reach and influence the developing fetus. These adaptations ultimately influence the total number of skeletal muscle fibers, peak bone mineral deposition, and the microstructure of articular cartilage [17].

### 2.2. Regulation of Embryonic Organ Development by Maternal Exercise and Nutrition

#### 2.2.1. Nervous System Development

During the early stages of nervous system construction, the central and peripheral networks exhibit highly complex and ordered developmental patterns. In terms of the central nervous system (CNS), its development initiates with the specific patterning of the neural plate [19]. Among these, neuromesodermal progenitors (NMPs), serving as a bipotent stem cell pool, play a crucial role in establishing connections with the posterior nervous system (lumbar, sacral, and caudal segments), and their developmental process demonstrates significant axial specification and spatiotemporal characteristics unique to primates [17]. Meanwhile, regarding the peripheral nervous system (PNS), neural crest cells, sensory neuron cells, and Schwann cell precursors construct a highly heterogeneous peripheral neural network through well-orchestrated migration and proliferation [20,21].

**Figure 1:**
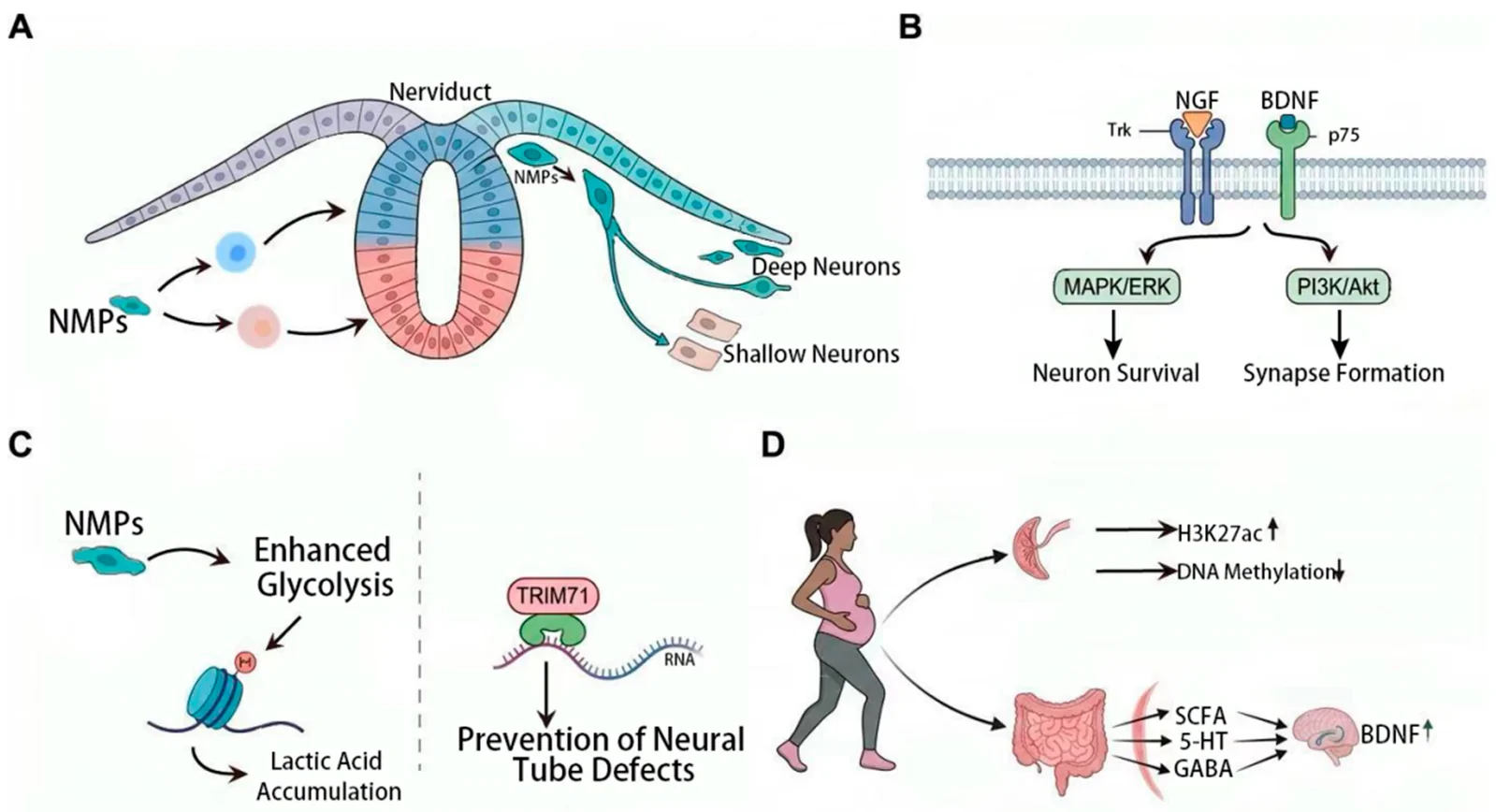
This figure depicts the comprehensive mechanisms underlying embryonic neural development and the positive impact of maternal environmental factors on fetal neurogenesis. It shows how (**A**) neuromesodermal progenitors (NMPs) drive the formation of the neural tube and differentiate into distinct lineages (deep and shallow neurons); (**B**) neurotrophin signaling via NGF/Trk and *BDNF*/p75 receptors activates the MAPK/ERK and PI3K/Akt pathways to ensure neuron survival and synapse formation; (**C**) metabolic reprogramming in NMPs (enhanced glycolysis and lactic acid accumulation) drives early developmental epigenetics, alongside TRIM71-mediated RNA regulation to prevent neural tube defects; and (**D**) maternal exercise orchestrates maternal–fetal crosstalk by inducing placental epigenetic shifts (upregulated H3K27ac, downregulated DNA methylation) and elevating gut-derived neuroactive metabolites (SCFA, 5-HT, GABA) to boost fetal brain *BDNF* levels, highlighting the intricate integration of cellular differentiation, metabolic cues, and maternal lifestyle in shaping the developing nervous system. Arrows indicate the direction of differentiation, signaling, or transport; colors distinguish cell types and pathway components. ↑ indicates increases and ↓ indicates decreases.

In fact, the aforementioned intersecting network comprising metabolic, epigenetic, and RNA regulation not only governs fundamental neurogenesis but also serves as highly sensitive molecular targets, bridging intrinsic developmental programs with extrinsic environmental interventions. Based on these targets, maternal exercise, as a critical environmental modifier, can play a pivotal protective role in promoting nervous system development and counteracting the effects of an adverse intrauterine environment by modulating maternal and fetal epigenetic networks and related molecular pathways [22,23]. At the molecular level, the balance between neural stem cell self-renewal and differentiation is tightly governed by the Notch and Hedgehog (Shh) signaling pathways [24]. Notch activation via Delta/Jagged ligands maintains the progenitor pool by upregulating Hes/Hey transcriptional repressors, while Shh from the notochord and floor plate establishes ventral patterning of the neural tube. The convergence of these pathways with metabolic cues (e.g., glycolysis-derived lactate and α-ketoglutarate) modulates histone acetylation and TET-mediated DNA demethylation, thereby influencing the expression of proneural genes such as *Neurog2* and *Ascl1*. Maternal exercise has been shown to fine-tune these signaling nodes in rodent models, shifting the balance toward enhanced neurogenesis rather than premature differentiation. Current evidence indicates that maternal exercise (rodent models and human correlational studies) not only promotes general neurogenesis but also exerts profound neuro-restorative effects and epigenetic reprogramming [25,26]. At the level of epigenetic remodeling, maternal exercise can induce widespread histone modifications (e.g., H3K27ac) and DNA methylation alterations (mouse study) in key brain regions such as the fetal hippocampus and placental tissues, thereby activating gene programs related to neurogenesis and synaptic plasticity. This mechanism can even reverse offspring epigenetic abnormalities triggered by adverse maternal metabolism (e.g., a high-fat diet) [23,27,28]. For example, maternal exercise has been shown to upregulate the expression of neuron-derived neurotrophic factor (*NDNF*) in the fetal hypothalamus and may repair neuronal depletion and axonal abnormalities caused by nutritional stress by modulating the abundance of specific microRNAs (such as *miR-505-5p*) (mouse model of gestational high-fat diet). Furthermore, at the systemic regulatory level, the neurological benefits of prenatal exercise partially depend on the mediation of the “gut–brain axis” [23,27,28,29,30]. Animal studies have verified that prenatal exercise can significantly optimize the composition of the maternal gut microbiota and elevate the levels of key metabolites, including short-chain fatty acids (SCFAs), serotonin (5-HT), and γ-aminobutyric acid (GABA) (mouse study). These metabolites can directly act on the fetus by crossing the placental barrier or by shaping the offspring’s initial microbiota, thereby upregulating *BDNF* expression in the offspring’s hippocampus (mouse model). This provides protective programming for the normal construction of the offspring’s nervous system and long-term cognitive function [25,31,32] (Figure 1).

In addition to exercise, maternal nutrition—particularly folate and omega-3 polyunsaturated fatty acids (PUFAs)—plays a synergistic role in fetal neurogenesis. Folate is essential for neural tube closure and DNA methylation dynamics [33], with prospective cohort evidence showing that continued folic acid supplementation during the second and third trimesters significantly reduces the risk of neurobehavioral developmental delays in early infancy [33,34]. Omega-3 PUFAs, especially DHA, are integral components of neuronal membranes and key regulators of synaptogenesis and neuronal survival. Emerging evidence suggests that combining maternal exercise with adequate intake of these nutrients may amplify the upregulation of *BDNF* and other neurotrophic factors, possibly through shared pathways involving gut microbiota metabolites, such as short-chain fatty acids (SCFAs), and epigenetic regulation [35,36]. The neurogenesis and synaptic plasticity processes described above are primarily governed by *BDNF*/TrkB signaling, Wnt/β-catenin pathways, and Notch-mediated progenitor maintenance. These pathways regulate neural stem cell self-renewal, neuronal differentiation, and synapse formation in the developing hippocampus and cortex, independent of the bone morphogenetic protein (BMP) signaling that predominantly governs skeletal development. Maternal exercise exerts its primary neurodevelopmental effects through enhanced placental perfusion, gut–brain axis modulation and *BDNF* upregulation. Selected nutrients—including folate, omega-3 PUFAs and dietary fiber—have been independently associated with offspring neurocognitive outcomes in separate cohort studies, and may potentially interact with these exercise-induced pathways. However, published studies directly testing combined exercise–nutrition interventions on fetal brain development are scarce, and the proposed additive or multiplicative effects remain speculative until such data emerge. Of note, the gut microbiota–exercise interplay described above is predominantly derived from mouse models in which exercise and diet were simultaneously manipulated. Human observational studies have reported correlations between maternal physical activity, gut microbial composition and cord blood SCFA levels, but causal evidence for the exercise–diet–microbiota–fetal outcome chain is lacking, and the relative contribution of exercise versus dietary substrates to circulating SCFA pools remains unquantified in pregnancy.

#### 2.2.2. Cardiovascular System Development

During the early construction of the embryonic cardiovascular system, the formation of the capillary network is primarily predicated on “vasculogenesis”—the in situ differentiation, migration, and assembly of mesoderm-derived angioblasts into an initial primitive plexus [37]. As the embryo grows rapidly to meet the exponentially increasing metabolic demands, this primitive network must undergo dramatic remodeling and expansion, a process known as “angiogenesis.” Angiogenesis is not merely an increase in vessel number but a master sequence of network functional specification. Under the joint guidance of molecular signals and hemodynamics, the primitive homogeneous vessels gradually specify, establishing a hierarchical system of arteries, veins, and capillaries with distinct functions [37,38,39,40,41]. Although the construction of the embryonic cardiovascular system follows inherent genetic programs, recent studies have revealed that heritable epigenetic regulation plays an indispensable spatiotemporally specific modulatory role in this process. Epigenetic mechanisms, without altering the DNA sequence, precisely control the expression levels of key cardiac transcription factors (e.g., *GATA4*, *Nkx2.5*, *Tbx5*) by dynamically remodeling chromatin structure and regulating transcriptional accessibility [42,43,44]. Crucially, epigenetic marks possess high environmental responsiveness, constituting the primary molecular interface through which maternal physiological states (such as exercise-induced metabolic changes) are biologically programmed into the fetal genome [45,46,47].

Beyond molecular regulators, hemodynamic forces—particularly laminar shear stress generated by fetal cardiac output—are critical for vascular network maturation. Shear stress activates the mechanosensitive Piezo1 channels and integrin-mediated FAK/ERK cascades, leading to upregulation of *KLF2* and eNOS, which in turn maintain an anti-thrombotic and vasodilatory phenotype. This mechanotransductive program also promotes endothelial cell alignment and arterial-venous specification via Notch and EphrinB2/EphB4 signaling. Maternal aerobic exercise, by enhancing placental blood flow and fetal heart rate variability, is hypothesized to augment these shear stress-dependent developmental cues, as supported by both rodent models and human Doppler studies. Current research demonstrates that (rat model of maternal hypertension), in terms of epigenetic and oxidative stress regulation, maternal exercise can significantly upregulate *SIRT1* expression in fetal blood vessels. Elevated *SIRT1* specifically induces deacetylation in the promoter region of the *Nox4* gene (spontaneously hypertensive rat model), leading to a significant reduction in histone H3K9ac levels, thereby inhibiting *Nox4* expression at the transcriptional level and reducing reactive oxygen species (ROS) generation. While exerting vascular protective effects, this mechanism significantly improves the local bioavailability of nitric oxide (NO) and endothelial-dependent vasodilation in the fetus [48]. In addition to antioxidant protection for the vascular system, maternal exercise also plays a key role in the epitranscriptomic remodeling of offspring cardiac development. In pathological models such as gestational diabetes mellitus (GDM), an intrauterine high-glucose environment can significantly disrupt the non-coding RNA regulatory network in the fetal rat heart, and maternal exercise has been proven to effectively reverse this adverse pathological programming. Specifically, exercise can significantly upregulate the expression level of a specific circRNA (*circ_0003226*). Acting as a competing endogenous RNA (ceRNA), *circ_0003226* (rat model of gestational diabetes mellitus) can specifically sponge excess *miR-351-5p*, thereby relieving the post-transcriptional inhibition of this miRNA on downstream target mRNAs. This mechanism effectively maintains the stability of target genes, ensuring the normal translation of key proteins required for cardiac development [49]. Given that circRNAs are characterized by high stability, evolutionary conservation, and tissue specificity, this finding not only uncovers a novel epitranscriptomic mechanism by which exercise protects offspring cardiac development but also provides potential targets for developing new molecular biomarkers to evaluate the efficacy of exercise interventions. Echoing the molecular mechanisms of the aforementioned basic research, clinical evidence further indicates that regular aerobic exercise during pregnancy can directly improve the hemodynamic state of the human fetal heart, specifically manifested by the significant optimization of core indices such as cardiac outflow tract blood flow velocity and ventricular filling parameters [50,51]. The vascular protective effects of maternal exercise are primarily mediated via *SIRT1*–*Nox4* epigenetic regulation and enhanced NO bioavailability. Dietary nitrate from vegetables has been shown to improve vascular function in non-pregnant populations and eNOS^−^/^−^ mouse models, and may potentially serve as an additional NO source during pregnancy. Nevertheless, human pregnancy studies have not directly tested whether exercise and dietary nitrate produce additive vascular benefits; the current evidence for synergy is therefore limited to indirect mechanistic inference.

Beyond exercise-induced *SIRT1*-mediated epigenetic regulation, maternal dietary nitrate intake represents a complementary nutritional strategy to further enhance NO bioavailability and potentiate the vascular protective effects of exercise. The nitrate-nitrite-NO pathway, abundantly present in dark leafy greens and beetroot, provides an alternative source of NO that bypasses the conventional L-arginine–eNOS pathway, which is often compromised under conditions of oxidative stress or endothelial dysfunction [52,53,54]. Mechanistically, dietary nitrate is reduced to nitrite by oral commensal bacteria and subsequently to NO via various reductase pathways in tissues, maintaining NO bioavailability even when eNOS activity is impaired [53]. In pregnant animal models, maternal beetroot juice (BRJ) supplementation has been shown to increase plasma nitrate and nitrite concentrations, lower systolic blood pressure, and improve uterine artery endothelial function, with effects partly independent of nitrate but nonetheless contributing to improved vascular health (mouse experimental study) [52]. Critically, NO precursor supplementation and exercise training have been demonstrated to exert synergistic effects on muscle function and vascular adaptations, with combination interventions yielding greater improvements than either modality alone (mouse experimental study) [55,56]. It should be noted that the nitrate-related vascular protective outcomes reported in this reference are derived from murine models rather than human clinical cohorts, clearly distinguishing preclinical animal evidence from human data. Extrapolating to the context of pregnancy, adequate maternal dietary nitrate intake—through consumption of nitrate-rich vegetables such as spinach, arugula, and beetroot—may serve to sustain the elevated NO production induced by regular physical activity, thereby amplifying the NO-mediated vasoprotection of the fetal vasculature. Thus, an integrated strategy of maternal exercise combined with sufficient dietary nitrate intake could more effectively preserve fetal cardiovascular health and counteract the adverse vascular programming induced by hypertensive or metabolic disorders during pregnancy.

#### 2.2.3. Digestive System Development

The construction of the digestive system is a developmental outcome of precise coordination among the three germ layers: the endoderm differentiates into the lining epithelium of the digestive tract, assuming the core functions of digestion and absorption; the mesoderm constructs the smooth muscle and vascular systems, providing the structural framework for dynamic contraction and blood circulation; and the ectoderm, through the colonization of neural crest cells, establishes the enteric nervous system (ENS), enabling local regulation of digestive tract functions [57]. The ontogeny of the digestive system is an extremely sophisticated multi-dimensional spatial construction process. The endoderm is the core functional origin of the digestive system, determining the efficiency of future digestion and absorption. During the 3rd to 4th week of cell growth, the endoderm forms the primitive gut tube (foregut, midgut, and hindgut) [58], and the epithelial primordia of the liver, pancreas, and gallbladder all develop from the ingrowth of the foregut endoderm into the surrounding mesenchyme [59]. Splanchnic mesoderm cells surround the endoderm-derived primitive gut tube and differentiate into the smooth muscle layer and submucosal connective tissue of the tract wall. Concurrently, the lateral plate mesoderm develops to form the mesentery and its internal rich vascular network [59,60]. The rhythmic mechanical load generated by spontaneous fetal movements is a key stimulus promoting the development of the aforementioned systems. This load can activate the RhoA/ROCK signaling pathway in smooth muscle precursor cells, upregulating contractile protein synthesis and laying the foundation for postnatal gastrointestinal peristalsis (rodent and avian embryonic models) [59]. The neural innervation of the digestive system originates from the ectoderm. A subset of ectodermal cells differentiates into neural crest cells, which possess high migratory and differentiation potential. Cranial neural crest cells migrate directionally along the rostrocaudal axis, ultimately colonizing the wall of the primitive digestive tract and differentiating into the key structures of the ENS—the submucosal and myenteric plexuses, allowing the ENS to independently regulate gastrointestinal functions [59,61,62].

**Figure 2:**
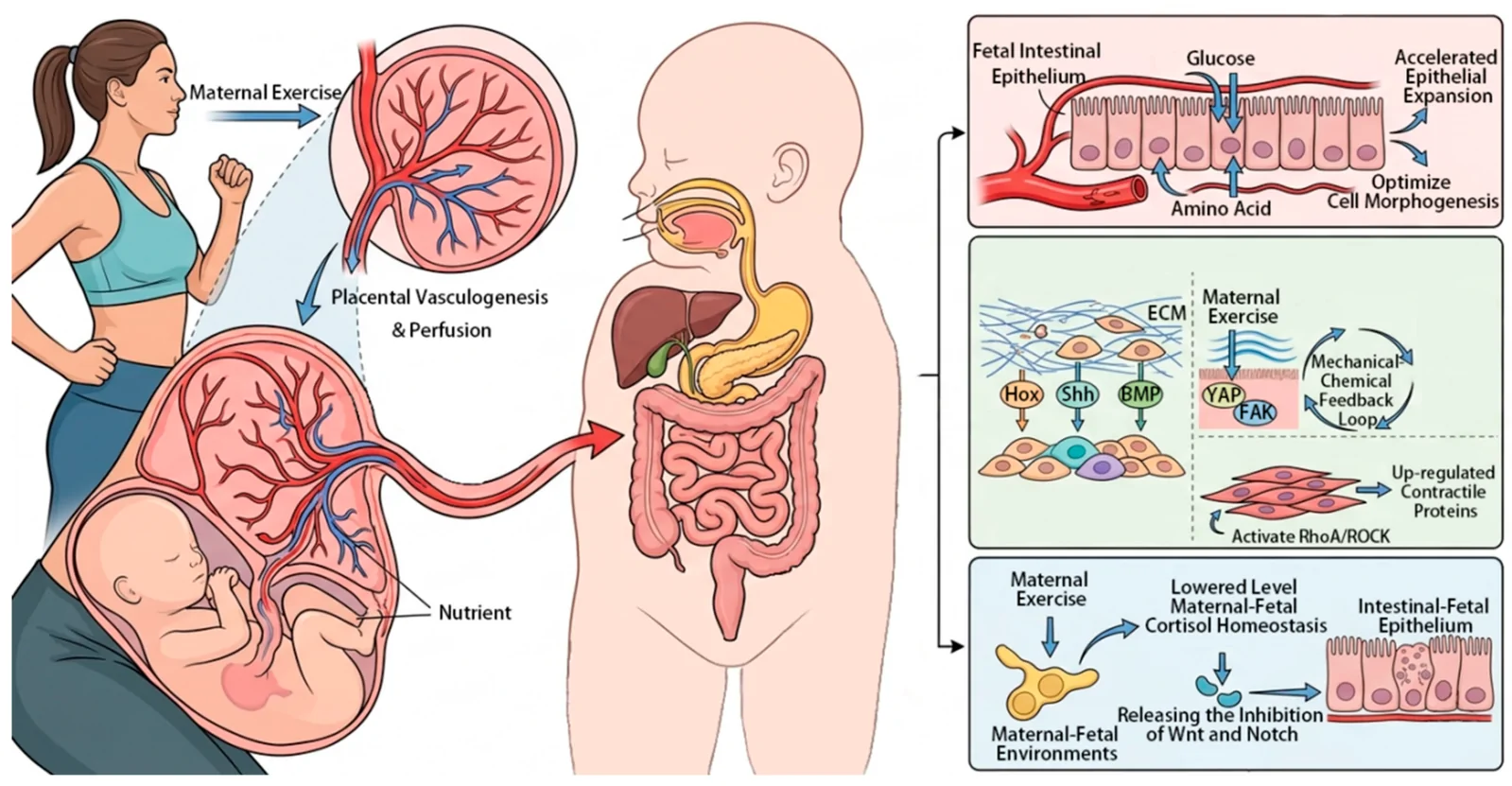
This figure depicts the comprehensive physiological and molecular mechanisms by which maternal exercise promotes fetal gastrointestinal development. It shows how maternal exercise initially enhances placental vasculogenesis and perfusion (**left**) to increase nutrient delivery to the fetus; concurrently, within the fetal gut (**right**), this increased nutrient supply (glucose and amino acids) drives accelerated epithelial expansion and optimizes cell morphogenesis (**top** panel); meanwhile, extracellular matrix cues (Hox, Shh, BMP) and exercise-induced mechanical-chemical feedback loops (YAP, FAK) activate the RhoA/ROCK pathway to up-regulate contractile proteins (**middle** panel); finally, lowered maternal–fetal cortisol homeostasis releases the inhibition of Wnt and Notch signaling pathways (**bottom** panel), highlighting the synergistic integration of enhanced nutrient transport, mechanotransduction, and endocrine modulation in shaping fetal intestinal maturation. Arrows indicate the direction of nutrient transport and pathway activation or inhibition; colors distinguish anatomical structures and signaling modules.

The development of the embryonic digestive system is highly sensitive to the intrauterine environment, and regular moderate-intensity maternal exercise can significantly promote the development and maturation of this system through multi-dimensional optimization of the intrauterine microenvironment. In terms of blood flow and nutrient supply, maternal exercise enhances placental angiogenesis and increases blood perfusion at the maternal–fetal interface. This hemodynamic improvement provides the fetus with more abundant oxygen and nutrient substrates (human placental studies and animal models), such as glucose and amino acids, thereby meeting the high metabolic demands of the primitive gut and pancreas during the rapid epithelial expansion phase and optimizing cellular proliferation and morphogenesis [57,63,64]. Beyond nutrient supply, the optimization of the intrauterine biomechanical microenvironment is another key pathway through which maternal exercise exerts its effects. During organogenesis, molecular signals such as Hox, Shh, and BMP dictate the initial mechanical properties (e.g., stiffness and anisotropy) of digestive tract tissues by guiding cell differentiation and extracellular matrix (ECM, e.g., elastin) deposition. Physical stimuli induced by maternal exercise (e.g., regular amniotic fluid fluctuations) can convert mechanical stress into biochemical signals by activating mechanosensitive pathways like YAP and FAK in fetal tissues, thereby retroactively regulating gene expression. This dynamic mechanical-chemical feedback loop not only dictates how cells respond to physical forces but also ensures the precise shaping of the complex structure and function of digestive organs, providing a solid biomechanical theoretical basis for the early programming of offspring gut health by prenatal exercise [57,65,66]. Moreover, at the endocrine regulation level, maternal exercise helps maintain maternal–fetal cortisol homeostasis. Existing research indicates that maternal exercise can reduce the cortisol burden in the maternal environment, thereby releasing the potential inhibition of high concentrations of glucocorticoids (rodent models) on key developmental signaling pathways such as Wnt and Notch. This improvement in the endocrine environment maintains the normal balance between proliferation and physiological apoptosis in embryonic intestinal epithelial cells, thereby providing a crucial safeguard for the healthy development of the embryonic gut [67] (Figure 2). Maternal exercise promotes fetal gastrointestinal development through mechanotransductive signaling, improved splanchnic perfusion and endocrine modulation. SCFA substrates from dietary fiber and vitamin D have been independently associated with enhanced intestinal barrier function and enteric nervous system maturation in animal models and human cohort studies, yet combined intervention data in pregnancy are lacking. Whether exercise and these nutrients yield coordinated benefits on fetal gut programming remains to be established.

Beyond maternal exercise, specific dietary nutrients play a complementary role in optimizing fetal gastrointestinal development. Maternal intake of dietary fiber—primarily fermented by gut microbiota into short-chain fatty acids (SCFAs) such as acetate, propionate and butyrate—has been shown to enhance intestinal barrier integrity by upregulating tight junction proteins and promoting mucin production [67]. Importantly, SCFAs can cross the placental barrier and exert direct effects on the developing fetal gut [68], where butyrate has been demonstrated to enhance the prenatal development of enteric neural crest stem cells and facilitate adult enteric neurogenesis, thereby contributing to the structural and functional maturation of the enteric nervous system (ENS) [69]. Emerging evidence also highlights the role of maternal vitamin D status in shaping the early-life gut microbiota of offspring, with deficiency during gestation being associated with altered microbial diversity, impaired intestinal barrier function, and increased susceptibility to inflammatory conditions in neonates [70]. Concurrently, adequate maternal intake of *n*-3 polyunsaturated fatty acids (PUFAs), particularly docosahexaenoic acid (DHA), has been shown to modulate the infant gut microbiome and mitigate Western diet-induced metabolic and neurodevelopmental impairments in offspring. Notably, the combination of exercise-induced hemodynamic optimization (enhancing nutrient delivery to the fetal gut) and adequate availability of SCFA precursors, vitamin D, and DHA may synergistically promote epithelial proliferation, maintain ENS integrity, and support the balanced establishment of the gut-associated lymphoid tissue. Therefore, an integrated strategy of maternal exercise combined with sufficient intake of fiber-rich foods, vitamin D, and *n*-3 PUFAs could more effectively promote healthy fetal digestive system development and reduce the risk of postnatal gastrointestinal disorders. Epithelial stem cells located at the base of the developing intestinal crypts are regulated by the Wnt/β-catenin gradient, which drives proliferation and transient amplifying cell expansion, whereas BMP signaling restricts stemness and promotes differentiation into absorptive and secretory lineages. The balance between these pathways is influenced by the biomechanical microenvironment and by metabolic substrates such as butyrate. Maternal exercise-induced fluctuations in amniotic fluid pressure and nutrient availability may modulate these pathways through epigenetic modifications at *Lgr5* and *Sox9* loci, as suggested by recent organoid and mouse studies.

#### 2.2.4. Respiratory System Development

The development of the respiratory system begins in the fourth week of human embryonic development, originating from the laryngotracheal groove, an outpouching of the ventral foregut endoderm [71]. With the formation of the longitudinal tracheoesophageal septum, the respiratory tract and the dorsal esophagus completely separate structurally and functionally, subsequently forming the independent larynx, trachea, and primary lung buds [70]. Following this, lung development sequentially undergoes key morphogenetic stages, including the pseudoglandular, canalicular, and saccular phases [72]. During this period, the lung buds construct a highly arborized bronchial tree network through a complex process of dichotomous branching. At the molecular level, this spatial patterning process is finely regulated by retinoic acid (RA) signaling [73,74]. RA not only guides the morphogenesis of airway branching and the construction of the vascular network through its crosstalk with FGF, Wnt, and other signaling pathways [72] but also precisely defines the differentiation patterns of the proximal airways and distal alveoli by inducing the spatiotemporally differential expression of transcription factors such as SOX2/SOX9. In the proximal airways, the epithelium primarily differentiates into pseudostratified ciliated columnar epithelium responsible for mucociliary clearance, mucus-secreting goblet cells, and supporting cells [75]. Conversely, in the distal environment filled with luminal fluid, a highly active progenitor cell population continuously proliferates, elongates outwards, and differentiates into respiratory bronchioles and alveoli, ultimately completing the architecture of the gas-exchange region [76]. Notably, abnormalities in the aforementioned RA signaling regulatory network are closely associated with severe fetal lung defects such as congenital diaphragmatic hernia (CDH). Distal lung progenitors express SOX9 and ID2, and their expansion is driven by FGF10 from the mesenchyme, while subsequent alveolar differentiation requires the sequential action of C/EBPα and PPARγ. Retinoic acid not only promotes branching but also induces the expression of surfactant proteins (SP-A, SP-B, SP-C) via RAR/RXR heterodimers. Maternal exercise, by improving uteroplacental oxygenation, may alleviate hypoxia-induced downregulation of these transcription factors, thereby preserving the normal developmental trajectory of alveolar type I and type II cells—a mechanism consistent with the reduced respiratory morbidity observed in human cohort studies [77,78].

**Figure 3:**
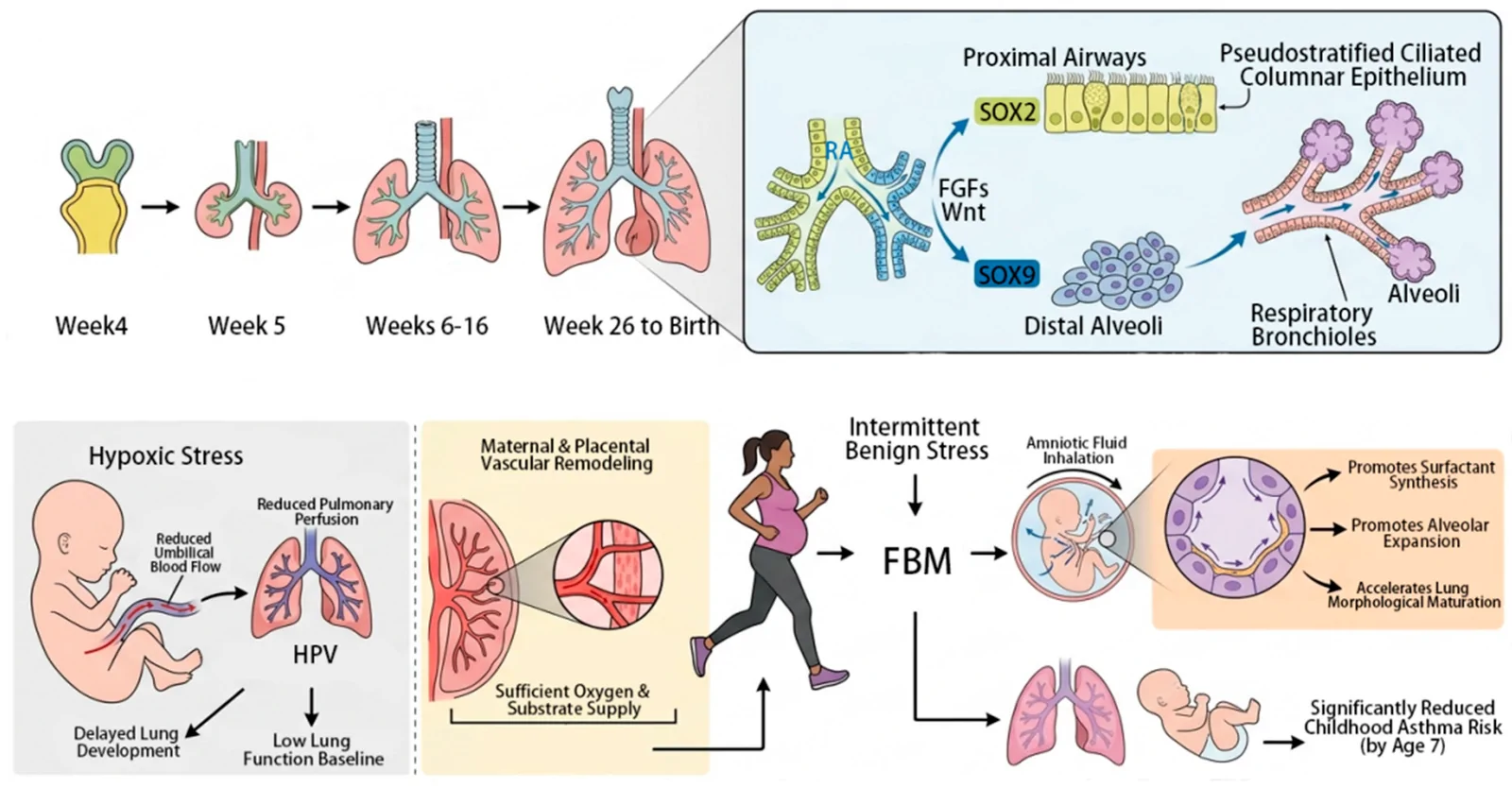
This figure depicts the spatiotemporal development of the fetal respiratory system and the contrasting impacts of hypoxic stress versus maternal exercise on lung maturation. It shows (**top**) the anatomical progression from week 4 to birth, where signaling gradients (RA, FGFs, Wnt) orchestrate branching morphogenesis, differentiating SOX2-expressing proximal airways (pseudostratified ciliated columnar epithelium) from SOX9-expressing distal alveolar progenitors; concurrently, it contrasts two physiological scenarios (**bottom**): hypoxic stress (**bottom left**) triggers hypoxic pulmonary vasoconstriction (HPV) and reduces perfusion, leading to delayed development and a low lung function baseline, whereas maternal exercise (**bottom right**) promotes placental vascular remodeling to ensure oxygen supply and acts as an intermittent benign stress. This exercise-induced stress enhances fetal breathing movements (FBM) and amniotic fluid inhalation, which mechanically drives surfactant synthesis and alveolar expansion, accelerating morphological maturation and ultimately correlating with a significantly reduced childhood asthma risk by age 7, highlighting the crucial interplay between genetic patterning, mechanical transduction, and maternal lifestyle in optimizing fetal pulmonary health. Arrows indicate developmental progression and directional physiological effects; colors distinguish developmental stages and the hypoxia and exercise conditions.

In response to these complex developmental processes, maternal exercise has been recognized as an effective non-pharmacological intervention strategy to promote the healthy development of the offspring’s respiratory system. Compared with other organs, the maturation of the fetal lung is highly dependent on oxygen and blood perfusion. Under intrauterine hypoxic stress, the fetus triggers a compensatory mechanism that redistributes blood flow to preferentially safeguard the blood supply to the heart and brain (the brain-sparing effect), leading to hypoxic pulmonary vasoconstriction (HPV). This causes a significant decrease in pulmonary perfusion, resulting in delayed fetal lung development due to inadequate nutrient and mechanical stimulation. However, regular moderate-intensity endurance training can promote maternal and placental vascular remodeling, increase resting umbilical blood flow (human studies and animal models), and enhance placental circulation efficiency, thereby providing ample oxygen and substrates for the high metabolism and rapid differentiation of the fetal lungs [77,79]. Although some studies indicate that acute exercise may lead to a transient attenuation of fetal breathing movements (FBM), this represents a compensatory physiological adaptation without long-term adverse effects [79]. In fact, clinical and epidemiological evidence suggests that maternal exercise exerts a long-term protective effect on the offspring’s respiratory system. Compared with sedentary pregnant women, infants born to mothers who engage in moderate exercise exhibit optimal baseline pulmonary function parameters at 3 months of age, with a significantly reduced risk of poor lung function. More importantly, regular maternal physical activity during pregnancy (≥3 times per week) significantly reduces the risk of offspring developing asthma during childhood (population-based birth cohort) (before age 7) [78], and this protective effect is independent of other potential confounding factors [79]. Regarding the underlying physiological mechanisms, we hypothesize that maternal exercise may act as an intermittent benign stressor that holistically enhances the frequency and intensity of FBM. The fluid expansion pressure generated by FBM-driven amniotic fluid inhalation can directly stimulate fetal alveolar expansion through mechanotransduction mechanisms, promote surfactant synthesis, and accelerate the morphological maturation of lung tissue (hypothetical mechanism inferred from rodent models and human observational data). This fundamentally optimizes the neonatal lung function developmental trajectory and reduces their susceptibility to childhood respiratory allergic diseases (Figure 3). The primary lung-protective effects of maternal exercise are linked to improved placental oxygenation, increased fetal breathing movements and mild oxidative preconditioning. Retinoic acid and vitamin D signaling independently support airway branching and immune maturation based on vitamin A/D deficiency and supplementation studies. However, no published pregnancy cohort or RCT has systematically evaluated the interaction between maternal exercise and vitamin A/D status on offspring respiratory outcomes; the proposed nutritional synergy is purely conjectural at this stage.

Beyond the beneficial effects of maternal exercise, adequate maternal nutrition plays a complementary and synergistic role in fetal lung development. The retinoic acid (RA) signaling pathway, which is essential for airway branching morphogenesis and alveolar differentiation, is highly dependent on maternal vitamin A status. Vitamin A deficiency during gestation impairs RA signaling, leading to abnormal lung bud patterning and reduced alveolarization, while sufficient maternal intake of provitamin A carotenoids and preformed vitamin A (retinol) supports normal RA-mediated lung development [74]. Emerging evidence also highlights the critical role of maternal vitamin D status in programming offspring respiratory health. Gestational vitamin D deficiency has been associated with an increased risk of childhood wheezing and asthma, likely through effects on fetal lung immune maturation, airway smooth muscle development, and surfactant production. Furthermore, oxidative stress is a known contributor to impaired fetal lung growth and increased susceptibility to postnatal respiratory diseases. Maternal intake of antioxidant nutrients such as vitamin C, vitamin E, and selenium can counteract oxidative damage to developing lung tissues, and these effects may synergize with exercise-induced upregulation of endogenous antioxidant enzymes such as superoxide dismutase (SOD) and glutathione peroxidase (GPx). Notably, the combination of regular maternal exercise (which enhances placental perfusion and fetal breathing movements) and adequate intake of vitamin A, vitamin D, and antioxidant micronutrients may collectively optimize the structural and functional maturation of the fetal respiratory system. Therefore, an integrated strategy of prenatal exercise combined with a nutrient-rich diet—including sufficient vitamin A, vitamin D, and antioxidants—could more effectively reduce the risk of childhood asthma and other respiratory morbidities.

#### 2.2.5. Musculoskeletal System Development

The development of the human skeletal system begins between the 6th and 8th weeks of gestation, primarily constructing the skeletal framework via two modes: intramembranous ossification and endochondral ossification [80]. This process is subjected to strict spatiotemporally specific regulation. Recent single-cell omics studies have unveiled the complex trajectories of mesenchymal progenitor cells differentiating into osteoblasts/chondrocytes, identifying specific cell subpopulations, such as Schwann cell precursors and Hic1+ progenitors, which play unique roles in the formation of the tendon-bone interface and the construction of fine skeletal structures [80,81]. Concurrently, the myogenesis of embryonic skeletal muscle is accompanied by the dynamic expression of myogenic regulatory factors such as *MyoD*, *MyoG*, and *Pax7*, which subsequently drive the rapid generation and maturation of muscle fibers [82]. Notably, the distal morphogenesis of osteoarticular structures is dictated not only by intrinsic developmental programs (e.g., WNT and BMP signaling) but is also highly dependent on the mechanical load generated by the embryo’s own movements [83]. Studies indicate that mechanical stress is crucial for the formation of the synovial joint cavity and the precise shaping of articular surfaces, whereas a lack of mechanical load leads to joint ankylosis or structural anomalies [83]. This suggests that physical activity by the mother or fetus during pregnancy may exert profound biomechanical effects on the early development of fetal joint cavities by altering the intrauterine hydrodynamic environment.

Maternal exercise synergistically promotes the development of the fetal musculoskeletal system primarily through three pathways: endocrine regulation, the transmission of myokines, and mechanical stimuli. First, at the endocrine level, research demonstrates that maternal exercise can significantly elevate triiodothyronine (T3) levels in the maternal and fetal systemic circulation. T3 can directly activate the transcriptional expression of these target genes by enhancing the binding of the thyroid hormone receptor (TR) to the promoter regions of myogenic regulatory factors (e.g., *MyoD*, *MyoG*), thereby promoting myoblast proliferation and myotube formation (mouse study) [84]. Second, regarding myokine transmission, exercise-induced myokines secreted by the mother can cross the placental barrier and activate the AMPK-*PGC-1α* signaling axis in fetal skeletal muscle. This process not only enhances mitochondrial biogenesis but also increases the proportion of oxidative muscle fibers, ultimately improving the metabolic flexibility of the offspring’s skeletal muscle (mouse model). Furthermore, maternal exercise can exert a profound early reprogramming effect on offspring skeletal muscle through epigenetic mechanisms. Exercise effectively prevents DNA hypermethylation in the promoter regions of key metabolic genes (such as *Ppargc1a*/*PGC-1α*) in offspring skeletal muscle caused by maternal obesity or a high-fat diet, thereby maintaining normal insulin sensitivity and muscle metabolic health in the offspring [85,86]. In pathological models (e.g., fetal akinesia deformation sequence, FADS), fetuses typically exhibit severe skeletal and joint developmental defects; however, recent studies have discovered that maternal exercise can partially compensate for the skeletal dysplasia caused by the lack of spontaneous fetal movement (mouse model of fetal akinesia) [87]. This further confirms that the mechanical stimuli or systemic circulating factors generated by maternal exercise play a crucial compensatory protective role in maintaining the normal morphogenesis of embryonic bones and joints.

Beyond mechanical and endocrine regulation, maternal nutrition interacts closely with exercise to shape fetal skeletal development. Adequate maternal intake of protein, calcium, phosphorus, vitamin D, and omega-3 fatty acids provides the essential building blocks for bone matrix formation and endochondral ossification. Dietary patterns such as the Mediterranean diet, rich in polyphenols and fiber, have been shown to modulate the maternal gut microbiota, increasing short-chain fatty acid (SCFA) production, which in turn enhances intestinal calcium absorption and reduces bone resorption. Importantly, exercise and nutrition exhibit synergistic effects: while exercise promotes mechanical loading and myokine release, including apelin and irisin, optimal nutrition ensures substrate availability for cartilage and bone anabolism. Preclinical evidence indicates that maternal diet–exercise interventions can rescue high-fat diet-induced impairments in offspring trabecular bone volume and cortical thickness (mouse study) [88,89]. Thus, integrating nutritional strategies with maternal exercise represents a more potent approach to optimizing fetal musculoskeletal programming under the DOHaD framework. Maternal exercise drives musculoskeletal programming through endocrine (T3/TR), myokine (Apelin–AMPK) and mechanical (YAP/FAK) pathways. Calcium, vitamin D and protein intake are well-established independent determinants of fetal bone mineralization in observational studies. Nevertheless, preclinical evidence for exercise–nutrition synergy on offspring bone remains limited to single mouse studies, and human data validating additive effects are absent. Caution is warranted in extrapolating combined benefits to clinical practice.

Beyond the organ-specific developmental outcomes discussed above, emerging evidence has focused on how maternal exercise modulates fetal body composition—particularly the accumulation of adipose tissue—without compromising overall growth [90]. A 2023 systematic review and meta-analysis of 63 randomized controlled trials (16,524 pregnant women) demonstrated that physical activity during pregnancy is significantly associated with a reduced risk of macrosomia (RR = 0.79, 95% CI: 0.63–0.98, *p* = 0.03), while no significant associations were found with birth weight, low birth weight, or being small/large for gestational age. This suggests that moderate prenatal exercise does not alter fetal birth weight or crown-heel length significantly, but rather selectively lowers excess fetal fat accumulation without impairing overall growth.

Supporting this distinction, a controlled exercise intervention study found that in utero exposure to exercise reduced infant mesenchymal stem cell lipid content (*p* = 0.01) and body fat percentage (*p* = 0.009) in a dose-dependent manner [91], with total exercise volume throughout pregnancy negatively correlated with infant body fat percentage (R^2^ = 0.31, *p* = 0.02), primarily due to lower subscapular skinfold thickness. Similarly, the prospective DALI cohort study (213 mother-child pairs with BMI ≥ 29 kg/m^2^) reported that higher maternal moderate-to-vigorous physical activity (MVPA) was associated with lower neonatal fat mass percentage in male offspring (−0.520%; 95% CI: −1.011%, −0.031%), with no associations found with birthweight. These findings collectively indicate that maternal exercise acts primarily on adipose deposition pathways—curtailing excess adiposity while preserving lean mass accretion—rather than on global growth restriction.

However, a critical nuance must be emphasized: physiological fetal fat reserves are indispensable for neonatal survival and adaptation [92]. The late third trimester witnesses the development of a deep layer of subcutaneous adipocytes rich in uncoupling protein 1 (*UCP1*), which are essential for non-shivering thermogenesis immediately after birth. This brown adipose tissue provides the primary heat source for newborns transitioning from the warm intrauterine environment to the extrauterine cooler milieu, and its thermogenic capacity is directly linked to neonatal survival. Furthermore, fetal fat stores serve as a critical energy reserve during the immediate postnatal period, when enteral feeding may be delayed or insufficient. As systematically reviewed by Ding et al. [93], maternal exercise contributes to improved glucose tolerance, reduced insulin concentration, and lower total cholesterol and LDL levels in adult offspring in mice—effects that are independent of maternal body weight and offspring dietary condition. This underscores that the benefits of maternal exercise on offspring metabolic health are not mediated simply by reducing fetal fat mass, but rather through broader metabolic programming.

Thus, the goal of maternal exercise is not to eliminate fetal fat but to prevent pathological excessive adiposity—the type associated with maternal obesity, gestational diabetes and subsequent childhood metabolic disease—while preserving the physiological fat depots required for normal neonatal metabolic adaptation. In practical terms, maternal exercise appears to reset the set point of fetal fat accumulation within a healthy physiological range. This nuanced perspective guards against the one-sided overstatement of fat reduction benefits and acknowledges that appropriate exercise balances fat accumulation—reducing the metabolic risks of overnutrition while safeguarding the adaptive reserves that support neonatal transition and early postnatal growth. Future research should focus on defining the optimal exercise dose that achieves this balance across diverse maternal metabolic phenotypes and fetal sex subgroups [92].

## 3. Regulation of the Maternal Microenvironment by Maternal Exercise

### 3.1. Remodeling of Maternal Metabolic Homeostasis by Maternal Exercise

Pregnancy is a unique and dynamic physiological window accompanied by maternal metabolic plasticity and immune adaptation, which are closely associated with maintaining maternal health and supporting fetal development [22,94]. In modern lifestyles, sedentary behavior during pregnancy increases the risk of maternal metabolic disorders, leading to elevated insulin resistance, dyslipidemia, and chronic low-grade inflammation [94,95], thereby exacerbating the incidence of gestational diabetes mellitus (GDM) and preeclampsia [96]. Regular maternal exercise represents not merely an increase in energy expenditure but a systemic physiological stimulus capable of progressively modulating maternal metabolic homeostasis through complex molecular networks.

Regarding the improvement of glucose metabolism and the reduction in GDM risk, regular metabolic exercise (particularly moderate-intensity aerobic exercise) can significantly activate maternal metabolic flexibility. This core adaptation is achieved through multiple pathways, including the regulation of branched-chain amino acid (BCAA) catabolism, the optimization of lipid oxidation efficiency, and the remodeling of the bile acid metabolic profile, thereby systematically ameliorating insulin resistance [97]. Mechanistically, exercise decreases circulating glucose levels and enhances fatty acid oxidation, which directly lowers blood glucose and amplifies systemic insulin sensitivity. This is of decisive significance for GDM prevention. Meta-analysis data indicate (human RCT meta-analysis) that structured prenatal exercise (e.g., moderate-intensity exercise for 30 min, three times a week) can significantly reduce the risk of developing GDM by approximately 43% [96]. Furthermore, this intervention is safe and does not result in low fetal birth weight, establishing maternal exercise as a first-line non-pharmacological strategy. In terms of specific metabolomic alterations, cutting-edge metabolomics-based studies have revealed that maternal exercise induces a beneficial shift in the profile of specific metabolites within the maternal circulation [95]. Exercise significantly reduces the circulating levels of leucine, isoleucine, and basic amino acids. Elevated levels of BCAAs are hallmark biomarkers of mitochondrial dysfunction and insulin resistance (acting via the hyperactivation of the mTORC1 signaling pathway to block insulin signaling). Exercise alleviates this blockade on insulin signaling (human and rodent studies) by promoting the oxidative degradation of BCAAs in skeletal muscle [98]. Moreover, exercise elevates the circulating levels of arginine and citrulline. As substrates for nitric oxide (NO) synthesis, the increase in these nutrients facilitates NO generation (human metabolomics and rodent mechanistic data), which not only improves vasodilation but also further promotes the uptake of nutrients such as glucose by skeletal muscles via enhanced blood perfusion [99].

Exercise corrects the endocrine imbalances of pregnancy by modulating the crosstalk between adipose tissue and skeletal muscle. Excessively high leptin levels during pregnancy typically indicate leptin resistance. Exercise optimizes energy balance signals by controlling excessive maternal body fat accumulation and maintaining leptin within a physiological range. Studies have demonstrated that low-to-moderate physical activity during late pregnancy is significantly and negatively correlated with maternal blood leptin levels, whereas sedentary behavior is directly associated with elevated pro-inflammatory cytokines (human cohort study) (such as IL-6 and TNF-α). The regulation of maternal adipokines by exercise primarily occurs through the modulation of amino acids and inflammatory markers. Exercise-induced IL-6, secreted by skeletal muscle as a myokine, exerts an anti-inflammatory effect; it inhibits TNF-α production and promotes the secretion of glucagon-like peptide-1 (GLP-1), thereby improving glucose metabolism and insulin secretion (human physiological study) [22]. Given that elevated leptin levels are typically associated with increased adiposity and insulin resistance, this indicates that maternal exercise can transform a pro-inflammatory environment into an anti-inflammatory one, conferring long-term protection for both maternal and fetal health. Maternal dietary composition profoundly influences these exercise-induced metabolic adaptations. For instance, a low-glycemic index diet or Mediterranean dietary pattern amplifies the glucose-lowering and insulin-sensitizing effects of exercise by modulating branched-chain amino acid (BCAA) catabolism and bile acid profiles. Protein intake quality and timing also affect the secretion of postprandial myokines, suggesting a diet–exercise interaction that collectively reduces gestational diabetes risk [10]. Regular maternal exercise independently reduces GDM risk (≈43% reduction in meta-analyses of exercise-alone RCTs) and improves systemic insulin sensitivity. Dietary patterns such as low-glycemic or Mediterranean diets have been separately validated as effective GDM prevention strategies in dietary intervention trials. However, direct head-to-head comparisons of exercise-alone versus combined diet-plus-exercise interventions are limited; two recent lifestyle RCTs reported inconsistent findings on additive effects, with one showing modest incremental benefit and the other demonstrating no significant difference from exercise alone. Consequently, the current evidence supporting synergy remains limited and model-dependent, precluding definitive conclusions.

### 3.2. Modulation of the Maternal Immune Microenvironment by Maternal Exercise

Pregnancy is not only a process of physiological structural remodeling but also a period during which the maternal immune microenvironment undergoes profound adaptive changes. To accommodate the fetus carrying paternal antigens, the maternal immune system must maintain a delicate balance between immune rejection and immune tolerance. However, the placenta is not an inert mechanical barrier but rather a highly dynamic immune signaling interface. As the direct contact point at the maternal–fetal interface, placental trophoblasts ubiquitously express various cytokine receptors (e.g., *TNFR*, *IL-6R*) and pattern recognition receptors (e.g., Toll-like receptors) on their surfaces, enabling them to acutely sense and respond to immune signals in the maternal circulation. Consequently, the maternal systemic immune status directly shapes the local placental immune microenvironment, transducing these states into biological cues perceivable by the fetus [100,101,102]. However, modern unhealthy lifestyles can easily disrupt this state of immune tolerance. With the rising obesity rates among women of reproductive age, chronic low-grade inflammation triggered by excessive visceral adiposity has emerged as a major obstetrical challenge [103]. Visceral fat, acting as an active endocrine organ, continuously secretes pro-inflammatory adipokines such as TNF-α and IL-6, which induce insulin resistance and disrupt immune homeostasis at the maternal–fetal interface. When the mother is chronically exposed to such inflammatory states (e.g., obesity or GDM), excessive pro-inflammatory cytokines can alter placental structure and function via four principal pathways: (1) Abnormal structural remodeling: inflammatory mediators induce trophoblast apoptosis, compromising the integrity of the placental barrier; (2) Vascular dysfunction: factors like TNF-α inhibit vascular endothelial growth factor (VEGF) signaling, interfering with placental angiogenesis and blood perfusion; (3) Impaired nutrient transport: inflammation downregulates the expression of glucose transporters (GLUTs) and amino acid transporters, directly compromising fetal energy supply; (4) Amplified oxidative stress: excess reactive oxygen species (ROS) activate local placental NLRP3 inflammasomes (human placental and rodent studies), further releasing pro-inflammatory cytokines such as IL-1β to create a vicious cycle. Ultimately, these pathological alterations severely perturb embryonic organogenesis and neural development [104,105,106].

**Figure 4:**
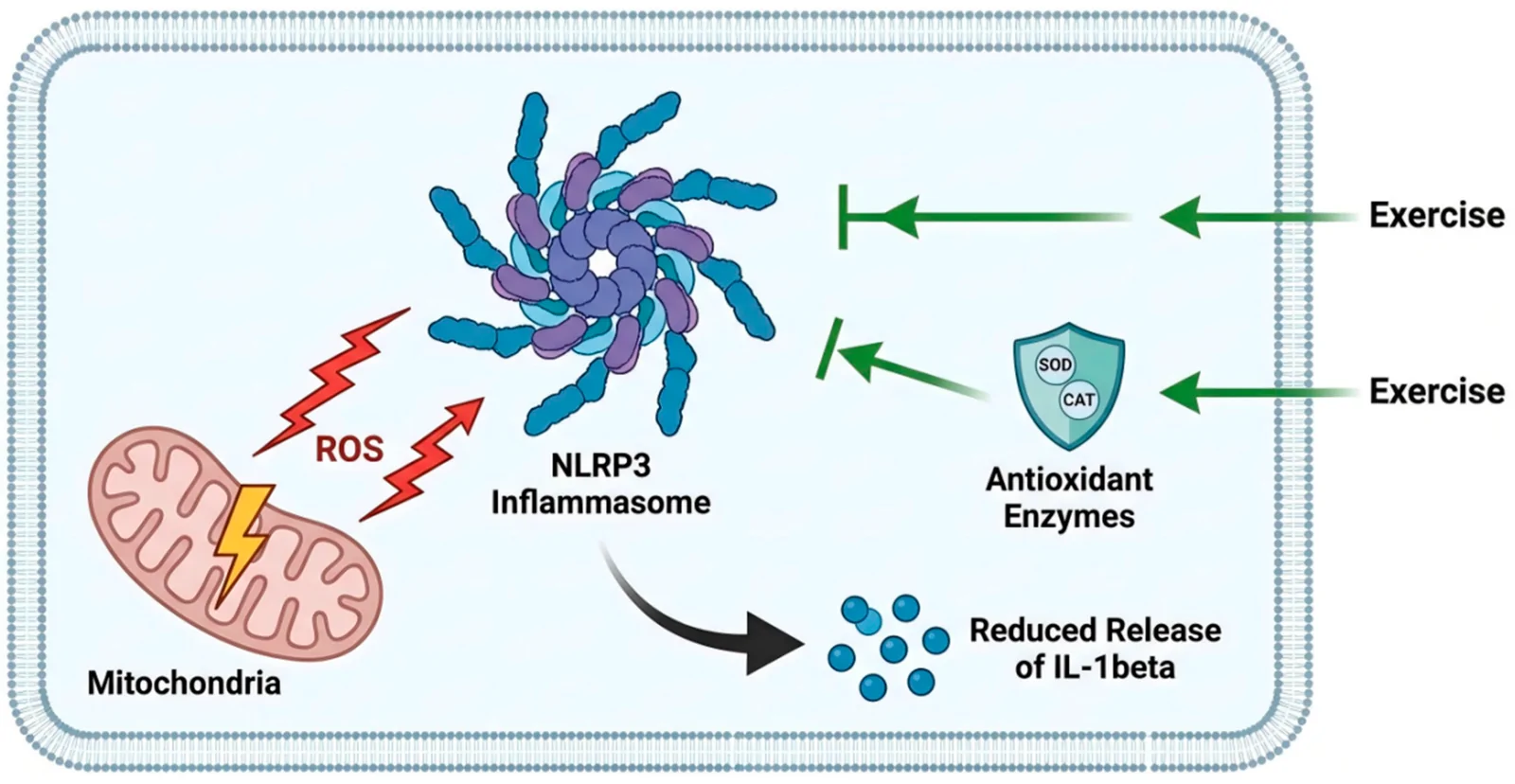
This figure depicts the intracellular mechanisms by which exercise exerts anti-inflammatory effects through the suppression of the NLRP3 inflammasome. It illustrates a dual-pronged protective pathway: exercise directly inhibits the assembly and activation of the NLRP3 inflammasome, while simultaneously up-regulating antioxidant enzymes (SOD and *CAT*) to scavenge mitochondria-derived reactive oxygen species (ROS). By neutralizing ROS, exercise effectively blocks the ROS-dependent trigger for inflammasome activation, collectively resulting in a significantly reduced release of the pro-inflammatory cytokine IL-1β, highlighting the critical role of physical activity in mitigating cellular oxidative stress and dampening inflammatory responses. Black arrows indicate ROS-dependent activation and downstream cytokine release; green blunt-ended lines indicate exercise-mediated inhibition, and green arrows indicate antioxidant-enzyme induction.

In response to these pathological challenges, regular maternal exercise has been recognized as a potent physiological immunomodulator. It not only controls maternal weight—thereby eliminating a primary source of pro-inflammatory cytokines—but also systematically reverses the maternal condition from a pathological pro-inflammatory state to a physiological anti-inflammatory protective state via active skeletal muscle contraction [107,108]. The core mechanism by which exercise regulates inflammation lies in the endocrine function of skeletal muscle. Modern physiology has established that contracting skeletal muscles release a series of bioactive substances termed “myokines,” which interact with organs such as adipose tissue and the liver to exert systemic anti-inflammatory effects [107,108]. Among these, the role of IL-6 is uniquely critical. Under resting conditions or within the adipose tissue of obese individuals, chronically elevated IL-6 is generally regarded as a pro-inflammatory marker. Conversely, exercise-induced, muscle-derived IL-6 exhibits a starkly opposite anti-inflammatory effect (human and rodent physiological studies). During exercise, the concentration of muscle-derived IL-6 increases transiently and markedly, without a concomitant rise in TNF-α [109], and robustly stimulates the release of anti-inflammatory cytokines, including IL-1ra and IL-10 [108,109]. This mechanism is particularly crucial for overweight pregnant women or those with GDM [110]. Exercise not only lowers the baseline levels of pro-inflammatory adipokines (e.g., leptin, resistin) by reducing visceral fat but also leverages skeletal muscle-derived IL-6 to rebalance pro- and anti-inflammatory cytokines, fundamentally reconstructing an anti-inflammatory microenvironment at a systemic level [108,109]. Beyond cytokine regulation, exercise-mediated inflammatory control also involves intracellular signaling pathways. In states of obesity and hyperglycemia, the intracellular multiprotein complex—the NLRP3 inflammasome—is easily activated, subsequently triggering Caspase-1 and promoting the maturation and secretion of potent pro-inflammatory cytokines like IL-1β and IL-18 [108,109]. Studies emphasize that exercise serves as an effective inhibitor of the NLRP3 inflammasome pathway (rodent models and human cell studies). By upregulating the activities of SOD and glutathione peroxidase, improving mitochondrial function, and boosting total antioxidant capacity, exercise effectively scavenges the excess ROS that incites inflammation [111] (Figure 4). This indicates that exercise not only reduces circulating inflammatory markers but also aborts the inflammatory cascade at its source, holding significant pathophysiological implications for mitigating gestational insulin resistance and preserving placental function [112].

Clinical cohort studies further substantiate the efficacy of these foundational mechanisms. Population-level data demonstrate that maintaining a high level of physical activity during pregnancy correlates significantly with lower circulating C-reactive protein (CRP) levels [111], confirming that regular exercise helps sustain a low systemic inflammatory burden [94,111]. Moreover, this systemic anti-inflammatory effect extends locally to the placenta. Exercise improves placental angiogenesis and mitigates tissue hypoxia and inflammatory infiltration, which not only optimizes the fetal nutritional microenvironment but also minimizes the risk of maternal vascular complications, such as preeclampsia, instigated by the systemic dissemination of placental inflammatory mediators [113]. In summary, via the intricate interface of the placenta, the maternal immune microenvironment profoundly influences and programs the embryonic developmental trajectory. Excessive chronic low-grade inflammation breaches immune tolerance, escalating the offspring’s long-term susceptibility to metabolic and neuropsychiatric disorders. Maternal exercise, serving as a non-invasive and multi-targeted intervention, systematically reshapes the immune microecology at the maternal–fetal interface by inducing muscle-derived anti-inflammatory myokines, suppressing NLRP3 activation, and alleviating oxidative stress, thereby securing a high-quality, low-inflammatory, and adequately perfused intrauterine environment for optimal embryonic development.

### 3.3. Regulation of Maternal Oxidative Stress Balance by Maternal Exercise

Oxidative stress refers to an imbalance between the production of reactive oxygen species (ROS) and the body’s antioxidant defense systems. An appropriate degree of oxidative stress facilitates trophoblast differentiation and invasion, supporting successful placental implantation and the establishment of the maternal–fetal circulation. However, excessive ROS production or inadequate antioxidant capacity can provoke pathological consequences, including vascular dysfunction, unchecked inflammation, and impaired fetal developmental programming. In normal pregnancies, the moderate ROS generated by the placenta acts not only as metabolic by-products but also as indispensable signaling molecules required for cellular signal transduction and gene expression regulation. Nevertheless, when the placenta is exposed to insults such as hypoxia, hyperglycemia, inflammation, or toxins, it produces an excess of ROS. A pathological state of high oxidative stress can precipitate extensive vascular endothelial damage and systemic inflammatory responses, acting as a core etiology for pathological conditions like preeclampsia (PE), gestational diabetes mellitus (GDM), and intrauterine growth restriction (IUGR). Recent studies indicate that maternal exercise can restore the oxidative balance within the maternal microenvironment through multifaceted biological mechanisms [114].

In terms of enhancing antioxidant defenses and the functional remodeling of high-density lipoprotein (HDL), maternal exercise initially combats oxidative stress by optimizing the maternal systemic metabolic state. While traditional perspectives focused solely on absolute lipid concentrations, recent research emphasizes the functional quality of HDL. Studies have identified a significant positive correlation between moderate-to-vigorous physical activity (MVPA) during pregnancy and the cholesterol efflux capacity (CEC) of HDL in maternal blood [115]. HDL not only mediates reverse cholesterol transport but also functions as a vital antioxidant carrier in the circulatory system, capable of neutralizing oxidized low-density lipoprotein (ox-LDL) and clearing lipid hydroperoxides. Evidence reveals that physically active pregnant women possess serum HDL with a superior capacity to clear accumulated cholesterol from vascular walls and withstand oxidative pressure. In contrast, sedentary behavior is associated with a decline in the activity of antioxidant enzymes (e.g., arylesterase/paraoxonase 1, PON1) [115]. This suggests that regular exercise establishes the mother’s primary defense against systemic oxidative damage by augmenting HDL functionality. Furthermore, the regulation of oxidative stress by exercise operates on the biological principle of hormesis. Although moderate exercise transiently induces minor ROS production in the short term, these ROS serve as signaling molecules that activate the body’s more robust endogenous antioxidant systems (such as *Nrf2* signaling, SOD, and GPx). This adaptive response substantially elevates both the baseline antioxidant capacity and the resilience against acute oxidative shocks in pregnant women who exercise regularly. Concurrently, it must be noted that excessive exercise may lead to ROS generation that surpasses clearance thresholds; therefore, identifying the optimal, individualized exercise volume is imperative [116].

Vascular endothelial dysfunction is the primary pathological alteration in hypertensive disorders of pregnancy, and oxidative stress is a key factor precipitating endothelial damage. Current research has thoroughly elucidated the molecular mechanisms by which exercise protects the vasculature. In the spontaneously hypertensive rat (SHR) model, maternal exercise has been demonstrated to significantly suppress the expression of NADPH oxidase 4 (*Nox4*) (spontaneously hypertensive rat model), a principal source of ROS generation within the vascular wall. This study revealed that exercise activates *SIRT1* (an NAD+-dependent deacetylase), causing the deacetylation of lysine residues (H3K9ac and H3K14ac) on histone H3 at the *Nox4* promoter region. This epigenetic modification represses the transcription of *Nox4*, thereby directly curtailing intravascular ROS production. This mechanism not only ameliorates maternal endothelium-dependent vasodilation but also enhances the activity of endothelial nitric oxide synthase (eNOS) and the bioavailability of nitric oxide (NO), providing a precise molecular target for the prevention of gestational hypertension. Beyond intracellular signaling pathways, exercise also promotes the secretion of exerkines from muscle and adipose tissues. Studies indicate that exercise can significantly elevate plasma Apelin levels (human and rodent studies) [117]. Beyond its roles in angiogenesis and metabolic regulation, apelin functions as a potent inducer of white adipose tissue (WAT) browning and brown adipogenesis—a process with particular relevance to fetal metabolic programming. Mechanistically, apelin activates the AMPK–*PGC-1α*–*UCP1* signaling axis, driving the expression of brown fat markers including uncoupling protein 1 (*UCP1*), *PRDM16*, and *Cidea* in fetal adipose tissue. In rodent models, maternal apelin administration during pregnancy increases brown adipose tissue (BAT) proteins such as *Cidea*, *Elovl3*, *UCP1*, *PRDM16*, and *PGC-1α* in both male and female fetuses, while concomitantly reducing white adipose tissue mass. This apelin-mediated browning program enhances mitochondrial biogenesis, oxidative phosphorylation, and thermogenic capacity in fetal adipose depots, establishing a metabolic phenotype that favors energy expenditure over storage. Notably, maternal exercise—which elevates circulating apelin levels—recapitulates these effects, suggesting that exercise-induced apelin upregulation serves as a key molecular link between maternal physical activity and favorable fetal adiposity programming. The relevance of this pathway to human fetal development is supported by observations that exercise and apelin increase thermogenesis and glucose uptake during pregnancy through activation of AMPK, PI3K, and *PGC-1α* signaling [22,118,119]. Apelin not only promotes angiogenesis but also specifically antagonizes the vasopressor effects of angiotensin II and mitigates oxidative stress by upregulating the expression of antioxidant enzymes. For populations at a high risk of preeclampsia, the exercise-induced increase in Apelin contributes to lowering the levels of anti-angiogenic factors, such as soluble fms-like tyrosine kinase-1 (sFlt-1), thereby improving placental perfusion and alleviating maternal vasospasm [118].

The oxidative state of the placenta directly dictates pregnancy outcomes. The placenta is not merely a passive organ for nutrient uptake but a highly active endocrine organ. Exercise can stimulate the placenta to secrete protective cytokines, such as superoxide dismutase 3 (SOD3), which is predominantly localized in the extracellular matrix. This ensures that extracellular matrix antioxidant enzymes can effectively scavenge superoxide in the intercellular space, erecting a barrier that protects fetal DNA from oxidative damage [5]. The exercise-induced upregulation of placental SOD3 acts as a pivotal barrier mechanism safeguarding the fetus against excessive maternal oxidative stress.

Placental development is highly reliant on energy provision from mitochondrial oxidative phosphorylation. In obese or diabetic pregnancies, placental mitochondria frequently generate copious amounts of ROS due to metabolic overload. Literature indicates that maternal exercise can promote placental mitochondrial biogenesis and functional adaptation, enhancing its oxidative metabolic efficiency and thereby minimizing ROS generation caused by electron leakage. However, another study focusing on obese pregnant women underscored the complexity of this mechanism. In severely obese populations, physical activity alone may be insufficient to completely reverse the accumulation of specific oxidative damage markers (human cohort of obese pregnant women) (such as 4-HNE or protein carbonyls) within the placenta [120]. This suggests that under conditions of profound metabolic perturbation, the ameliorative impact of exercise on local tissue oxidative stress may manifest a threshold effect or be counteracted by the interference of other inflammatory factors. Clinically, this implies that obese pregnant women may require higher-intensity exercise or concurrent dietary interventions to achieve a significant improvement in placental oxidative status.

**Figure 5:**
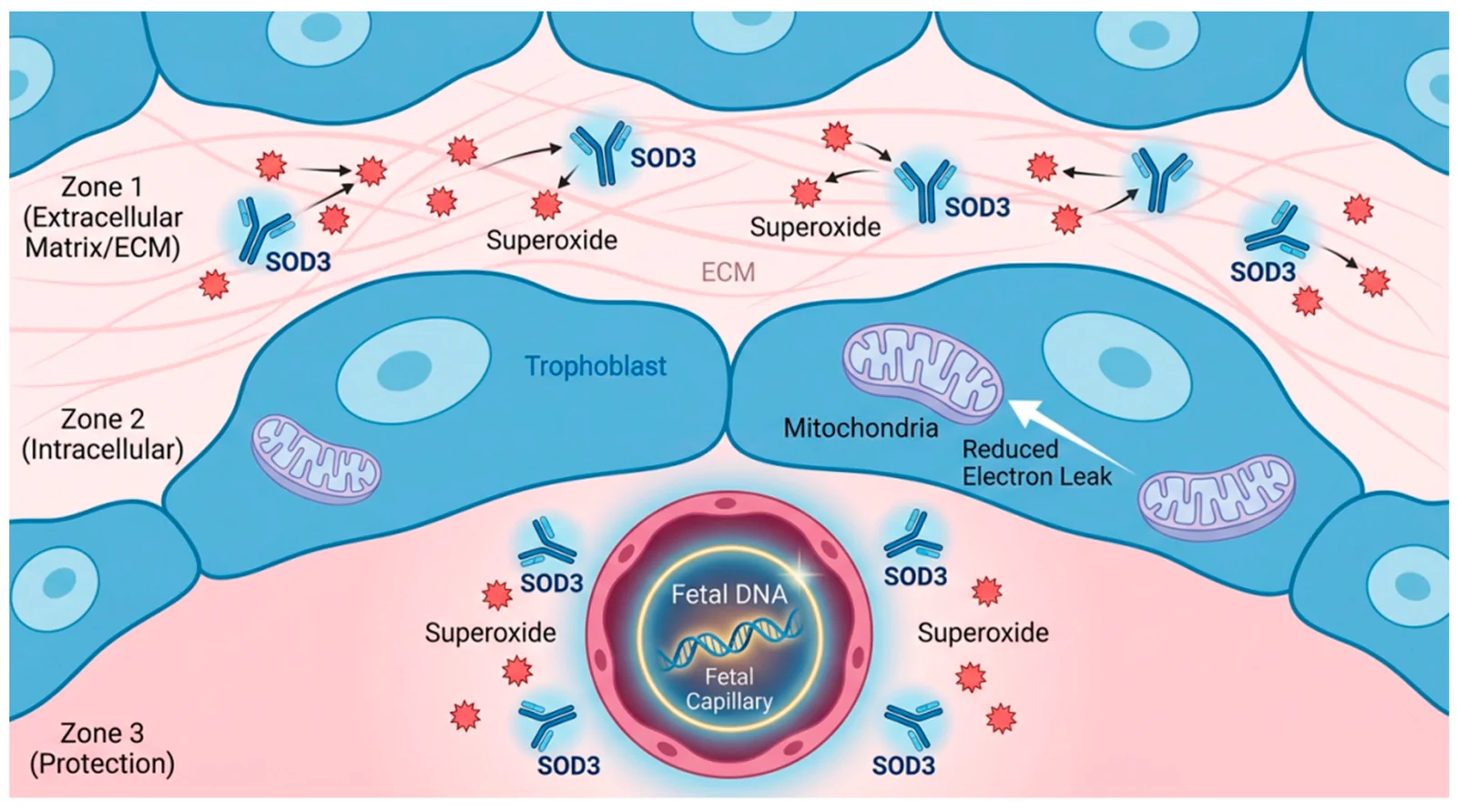
This figure depicts the multi-zonal antioxidant defense mechanisms in the placental microenvironment, emphasizing the protective role of Superoxide Dismutase 3 (SOD3). It illustrates a three-tiered spatial coordination: in Zone 1 (Extracellular Matrix/ECM), secreted SOD3 actively scavenges and neutralizes superoxide radicals; in Zone 2 (Intracellular), trophoblast cells exhibit enhanced mitochondrial efficiency characterized by reduced electron leak, which minimizes intrinsic reactive oxygen species production; and in Zone 3 (Protection), the concentrated presence of SOD3 around the fetal capillary creates a robust antioxidant shield, effectively neutralizing surrounding superoxide to safeguard fetal DNA from oxidative damage. This highlights the crucial integration of extracellular enzymatic defense and intracellular mitochondrial homeostasis in preserving placental integrity and protecting fetal genetic material. Note: Arrows indicate SOD3-mediated superoxide scavenging and reduced mitochondrial electron leak; colors distinguish the extracellular, intracellular, and fetal-protection zones.

In addition to isolated exercise, studies have also explored the impact of physical therapy—specifically, visceral manual therapy combined with physical activity—on maternal somatic pain and oxidative stress during pregnancy. Due to the enlarging uterus, the diaphragm and viscera undergo mechanical compression, which can easily induce local tissue hypoxia and ROS accumulation, consequently triggering symptoms such as lower back pain. Research demonstrates that this comprehensive intervention can enhance tissue microcirculation and accelerate the clearance of metabolic waste and oxidative byproducts [121]. This combined therapy is deemed beneficial for attenuating oxidative stress and alleviating tissue inflammation and pain driven by oxidative imbalance, thereby offering novel perspectives for prenatal care. In summary, the regulation of the maternal microenvironment and oxidative stress balance by maternal exercise constitutes a multi-layered, multi-targeted systemic network. Although its modulatory efficacy under specific pathological conditions (e.g., severe obesity) necessitates individualized consideration [120], the current body of evidence robustly supports moderate exercise as a core strategy for ameliorating the gestational oxidative stress state and preventing adverse pregnancy outcomes (Figure 5).

## 4. Regulation of Placental Function by Maternal Exercise

### 4.1. Impact of Maternal Exercise on Placental Morphology and Structure

The placenta, serving as the sole hub for maternal–fetal substance exchange, gas transport, and immune protection, directly determines pregnancy outcomes and the long-term health of the offspring. With the rise of the DOHaD hypothesis, the impact of maternal lifestyle during pregnancy, particularly physical activity, on the intrauterine environment has garnered increasing attention. Existing epidemiological evidence has confirmed that regular maternal exercise can significantly reduce the risks of gestational diabetes mellitus (GDM), preeclampsia, and macrosomia. However, the exact mechanisms by which exercise affects the placenta remain incompletely understood. Based on recent histological and molecular biological studies, we systematically elucidate the regulatory mechanisms of maternal exercise on placental morphology and structure from three dimensions: macroscopic morphology, microscopic tissue structure, and vascular network construction, and propose an exercise-induced “functional efficiency enhancement” hypothesis.

Early perspectives on the impact of maternal exercise on the placenta tended to assume that maternal exercise would compete for blood flow directed to the placenta, thereby resulting in reduced placental volume or weight. However, recent systematic reviews and multiple cohort studies indicate that this view is not supported by morphological evidence [122,123]. At the macroscopic level, there are typically no significant differences in placental weight, volume, or the placental-to-fetal weight ratio between exercising and sedentary pregnant women (human cohort systematic review and meta-analysis) [122]. This suggests that normal-intensity maternal exercise does not cause pathological compensatory hypertrophy or ischemia-induced atrophy in the placenta. This morphological stability is crucial; it implies that exercise does not disrupt the fundamental growth trajectory of the placenta [123]. At the physiological adaptation level, optimal fetal growth and development (e.g., appropriate birth weight and lower body fat percentage) are better supported without altering placental size. This indicates that exercise-induced adaptations manifest not as a simple physical expansion of organ volume, but as a systemic enhancement of cellular functional efficiency. The placenta meets the demands of exercise-induced maternal metabolic changes and fetal development through internal structural optimization rather than external volumetric enlargement.

Although macroscopic morphology remains stable, from a microscopic perspective, the exercised placenta exhibits significant structural remodeling. Current studies on obese pregnant women provide valuable microscopic insights. Research has found that moderate-to-vigorous physical activity (MVPA) during pregnancy is positively correlated with the volume density of placental villi (human cohort of obese pregnant women). Villi are the fundamental morphofunctional units for placental substance exchange; an increase in their density directly translates to an expanded maternal–fetal exchange surface area [124]. For obese pregnant women, the placenta often faces a risk of functional impairment due to chronic inflammation and oxidative stress, whereas exercise triggers this protective compensatory mechanism. Such microstructural alterations can be viewed as spatial optimization within the placenta. By increasing the number of villi per unit volume, the placenta’s capacity for oxygen and nutrient uptake and transport is significantly enhanced. This structural reserve capacity not only helps offset the potential adverse effects of maternal obesity on the intrauterine environment but also ensures an adequate nutritional supply during peak fetal growth. This adaptive remodeling demonstrates the high plasticity of the placenta, which is capable of sensing maternal mechanical and metabolic signals to make structural adjustments.

**Figure 6:**
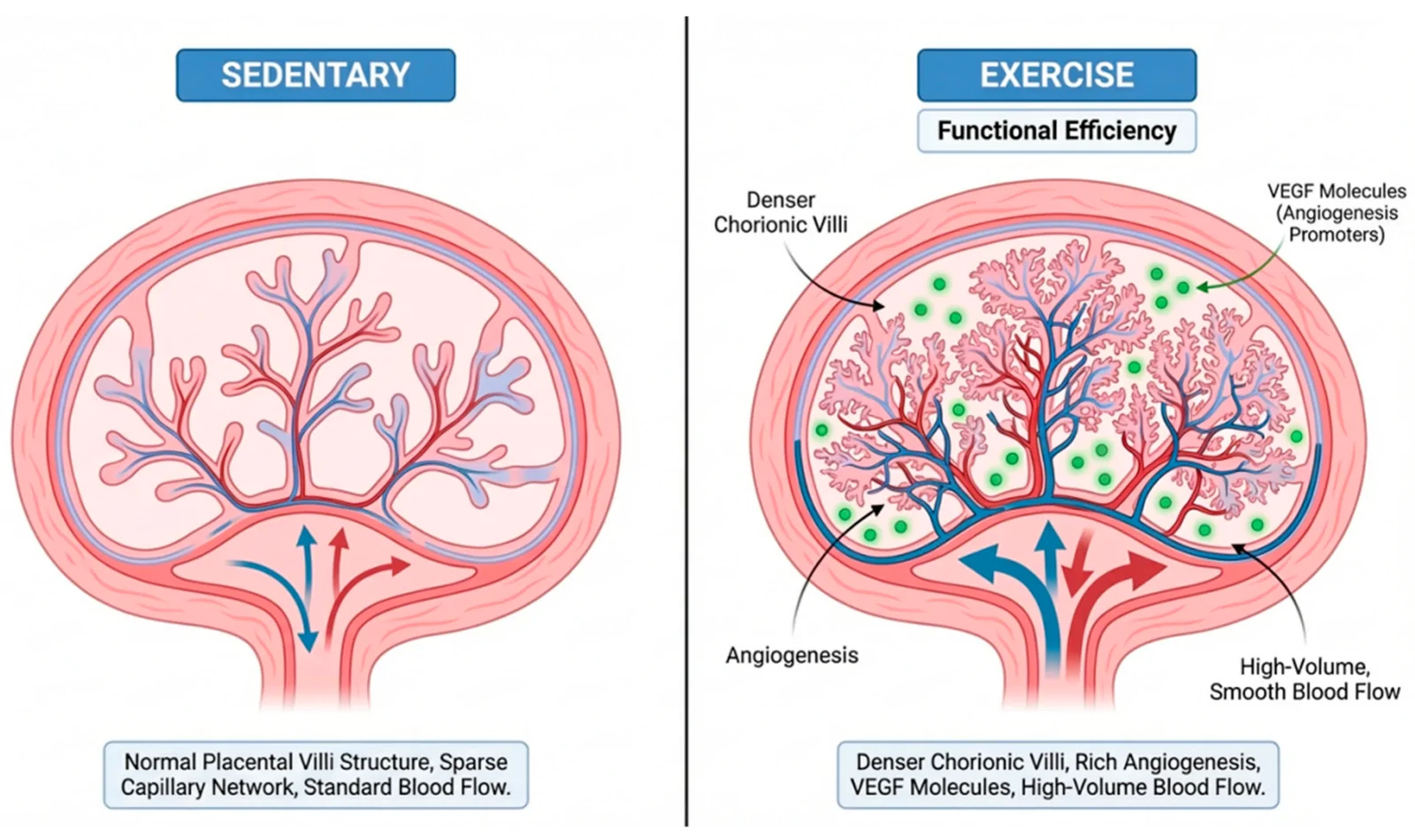
This figure depicts the impact of maternal exercise on placental morphology, angiogenesis, and overall functional efficiency compared with a sedentary state. It illustrates a clear structural and functional contrast: the placenta in the sedentary group (**left**) exhibits a standard chorionic villi structure with a relatively sparse capillary network and baseline maternal–fetal blood flow. Conversely, the exercise group (**right**) demonstrates significant physiological enhancements. Exercise induces the accumulation of Vascular Endothelial Growth Factor (VEGF) molecules, which act as robust angiogenesis promoters. This targeted up-regulation leads to richer angiogenesis and denser chorionic villi, thereby facilitating a high-volume, smooth blood flow. These adaptations highlight the critical role of physical activity in optimizing placental vascular architecture and functional capacity to robustly support fetal development. Red and blue arrows indicate opposing blood-flow directions, green dots denote VEGF molecules, and the left and right panels distinguish the sedentary and exercise conditions.

Vascular development is the core of placental function, and adequate blood perfusion is fundamental for fetal survival. Current research reveals how maternal exercise drives the construction of the placental vascular network at the molecular level. Studies show that the protein expression levels of vascular endothelial growth factor (VEGF) and its receptor (VEGFR-1) are significantly upregulated in the placentas of exercising pregnant women [125]. VEGF is a recognized key regulator of angiogenesis; its increased expression directly promotes the sprouting and branching of capillaries within the placenta. This pro-angiogenic environment yields structural benefits in both vascular density and vascular remodeling. Regarding vascular density, it forms a more compact capillary network, shortening the diffusion distance between maternal and fetal blood and enhancing oxygen diffusion efficiency. Regarding vascular remodeling, the hemodynamic shear stress generated by exercise acts as a physical signal, stimulating endothelial cells to release vasoactive substances, thereby maintaining the placental vascular bed in a state of low resistance and high flow. Such vascular adaptive changes hold significant clinical importance for preventing diseases associated with placental hypoperfusion (such as preeclampsia and fetal growth restriction). By establishing a more robust vascular network, prenatal exercise ensures that even during maternal physical activity, the placental circulation maintains stable perfusion pressure, preventing fetal hypoxia (Figure 6).

Notably, recent animal model studies reveal a potential sexual dimorphism in placental adaptation to exercise (mouse study). Placentas carrying male versus female fetuses may activate distinct gene expression pathways and structural remodeling patterns in response to maternal exercise [119]. Male placentas often exhibit higher sensitivity to growth-promoting signals; for example, the activity of their insulin-like growth factor (IGF) signaling pathway may be stronger, tending to prioritize resources to support rapid fetal somatic growth. Conversely, female placentas may demonstrate superior stress adaptation and metabolic reserve capacities. For instance, their expression profiles of antioxidant defense systems, endoplasmic reticulum stress response pathways, and glycogen metabolism-related genes may be more advantageous, aiding in maintaining fetal survival and neural development under nutritional or oxidative stress challenges. This discovery provides a novel theoretical basis for formulating personalized prenatal exercise prescriptions and highlights an important and novel direction for future research. This sexual dimorphism in placental adaptation is underpinned by several interconnected mechanisms. First, sex chromosome complement directly influences trophoblast proliferation and endocrine function: the X-linked genes (e.g., *Ogt*, *Eif2s3x*) modulate O-GlcNAcylation and mitochondrial metabolism, while Y-chromosome genes (e.g., *Sry*, *Uty*) may affect androgen signaling and vascular reactivity. Second, placental epigenetic landscapes differ intrinsically between male and female fetuses, with male placentas exhibiting lower global DNA methylation and higher expression of growth-promoting imprinted genes (e.g., *Igf2*), which may predispose them to greater responsiveness to exercise-induced growth stimuli. Third, sex hormone receptor distribution—particularly androgen receptor (AR) and estrogen receptor (ER) expression in trophoblasts and stromal cells—creates differential sensitivity to maternal circulating steroid hormones that are altered by exercise (e.g., cortisol, estradiol). Fourth, exerkine responsiveness may be sex-biased: apelin and irisin receptors (APJ and integrin αV/β5) show differential expression levels or downstream signaling kinetics in male versus female placental tissues, leading to divergent angiogenic and metabolic outputs. Finally, SCFA metabolites activate GPR41/43 with potential sex-dependent efficiency due to variations in receptor density or coupling affinity, though this remains largely unexplored in human placentas. Collectively, these mechanisms explain why male placentas often prioritize resource allocation for rapid somatic growth, whereas female placentas favor structural resilience and metabolic reserve—an evolutionary strategy that may confer survival advantages under nutritional or oxidative challenges.

These findings robustly support the inclusion of moderate exercise as an essential component of prenatal care. Exercise not only strengthens the mother but also, by remodeling the structure of the placenta—the vital hub of life—constructs an intrauterine developmental environment for the fetus characterized by richer blood supply, more efficient nutrient exchange, and a more stable metabolism [126]. Future studies should further explore the specific impacts of varying exercise intensities, frequencies, and initiation times on regional placental structures to provide more precise intervention strategies for improving pregnancy outcomes.

### 4.2. Regulation of Placental Nutrient Transport Function by Maternal Exercise

The placenta is the core organ for substance exchange, gas transport, and the immune barrier between the mother and fetus during pregnancy. Recently, the DOHaD theory has emphasized the deterministic role of the intrauterine environment on the offspring’s long-term health. Regular maternal exercise, as a non-pharmacological intervention, has been proven to ameliorate maternal metabolism and promote appropriate fetal growth, though its precise molecular targets remain incompletely elucidated. Current evidence indicates that maternal exercise finely regulates placental function by upregulating the expression of key nutrient transport proteins, optimizing mitochondrial bioenergetics, promoting angiogenesis, and mediating the crosstalk of “muscle-placenta” factors [127].

In the complex physiological process of pregnancy, the placenta, as a highly active metabolic and endocrine organ, dynamically adjusts the allocation of nutrients based on maternal nutritional status and fetal demands. Previous research largely focused on the impairment of placental function in pathological states (e.g., GDM, preeclampsia), often neglecting the shaping role of active lifestyle interventions, particularly prenatal exercise, on placental physiological functions. With the advancement of perinatology, accumulating clinical and basic research confirms that moderate prenatal exercise (whether acute single bouts or chronic regular training) can induce significant adaptive changes in the placenta [126,128]. These changes extend beyond simple increases in blood flow, involving the deep remodeling of gene expression, post-translational modifications of proteins, and organelle function. In-depth investigation of this process is of significant importance for preventing macrosomia, fetal growth restriction (FGR), and improving the metabolic health of the offspring [5].

Placental transport of glucose, fatty acids, and amino acids relies on specific membrane transport proteins. Exercise potentially modulates the expression and localization of these proteins in a substrate-specific manner.

Lipids are indispensable raw materials for fetal nervous system development and cell membrane construction. Current studies show that prenatal exercise is closely associated with the increased expression of placental fatty acid transport protein 4 (FATP4). FATP4 is predominantly localized on the microvillous and basal membranes of the placental syncytiotrophoblast, responsible for the uptake of long-chain polyunsaturated fatty acids (LC-PUFAs). Studies have found that FATP4 protein levels in the term placentas of the exercise group were significantly higher than those in the sedentary group (human cohort) [128]. Therefore, we postulate that exercise may promote the transcription and translation of FATP4 by activating the peroxisome proliferator-activated receptor (PPAR) signaling pathways. This implies that exercise enhances the placenta’s capacity to deliver critical fatty acids (such as DHA and EPA) to the fetus—which is crucial for fetal brain and retinal development—without causing excessive fetal fat accumulation. This demonstrates that prenatal exercise optimizes nutrient allocation efficiency rather than simply increasing total nutrient delivery [126,128].

Glucose is the primary energy source for fetal growth, crossing the placental barrier mainly through the facilitated diffusion carrier glucose transporter 1 (GLUT1). Regarding the impact of exercise on GLUT1, existing literature reports some discrepancies. Some studies indicate that chronic exercise can increase total placental GLUT1 protein expression (rodent and human placental studies, with discrepancies noted); however, other data suggest that while drastic changes in total expression were not observed, alterations in GLUT1 localization on the cell membrane or enhancements in transport efficiency were noted [128]. Reviews also point out that the effect of exercise on glucose transporters may depend on the maternal metabolic state [126]. Nevertheless, prenatal exercise undeniably exerts a regulatory function on placental glucose transport. Notably, in obese or hyperglycemic pregnant women, GLUT1 is often overexpressed, leading to macrosomia. Exercise interventions ameliorate this condition; under a maternal hyperglycemic environment, exercise improves insulin sensitivity, preventing the placenta from prolonged exposure to high glucose, thereby averting the pathological upregulation of GLUT1 and maintaining appropriate glucose flux [5].

Beyond carbohydrates and lipids, amino acid transport (e.g., System A/SNATs) is tightly regulated by the mTOR signaling pathway, which is decisive for fetal lean body mass accrual. Although studies directly addressing the regulation of specific amino acid transporters by exercise are currently limited, exercise has been proven to modulate the activity of the placental mTOR pathway (mouse model of fetal growth restriction). Collectively, maternal exercise constructs a metabolically favorable intrauterine environment by upregulating key transporters (FATP4) and maintaining the homeostasis of glucose transporters (GLUT1) [128].

### 4.3. Regulation of Placental Exosome Function by Maternal Exercise

Placenta-derived exosomes (30–150 nm in diameter) are core information carriers for maternal–fetal signal communication [129]. They are generated by syncytiotrophoblast cells via the endosomal pathway and are continuously released into the maternal peripheral circulation from the 6th week of gestation. Their release volume increases with gestational age, serving as real-time indicators of placental functional status. The mRNA, miRNA, proteins, and metabolites enriched within these exosomes exhibit source-cell specificity. Protected by their lipid bilayer, they can travel long distances within the maternal circulation to deliver metabolic, immune, and vascular regulatory signals [130,131,132]. This mechanism offers a novel molecular communication perspective on how prenatal exercise transmits beneficial biological effects to the mother and fetus via the placenta. Specifically, maternal exercise, as a systemic physiological stimulus, may reshape the metabolic and epigenetic state of trophoblast cells by altering the maternal circulating endocrine microenvironment. This subsequently regulates the release quantity and cargo microarray of placental exosomes, ultimately transmitting exercise-mediated adaptive information in the form of vesicles throughout the maternal–fetal system to achieve multi-system health programming.

Prenatal exercise regulates placental exosomes via the myokine network, a process heavily reliant on skeletal muscle-placenta crosstalk [118]. Exercise induces maternal skeletal muscle contraction, releasing various myokines (such as Apelin and Irisin). Once entering the bloodstream, these signaling molecules act as crucial upstream signals regulating placental exosome assembly [130,131,132]. Research indicates that exercise-upregulated Apelin can bind to placental APJ receptors, activating the AMPK/mTOR pathway (mouse study) to optimize glucose metabolism in trophoblast cells, and improving local placental blood flow via the eNOS/VEGF pathway [118]. Concurrently, Irisin can enhance antioxidant enzyme activity via the *Nrf2* pathway, mitigating local oxidative stress [118]. This optimization of placental cellular metabolism and alleviation of oxidative stress, mediated by myokines, directly dictate the sorting and assembly characteristics of exosomal cargoes. In a healthy trophoblast microenvironment characterized by low stress and maintained energy homeostasis, the placenta preferentially sorts and enriches molecules with vasoprotective and metabolism-promoting functions (e.g., specific miRNAs and anti-inflammatory proteins) into exosomes, rather than releasing pathological vesicles carrying damage or inflammatory signals [22].

In addition to myokines, the remodeling of the adipokine network by prenatal exercise is another critical pathway for regulating placental exosomes. Excessive maternal fatty acid accumulation during pregnancy leads to the abnormal elevation of pro-inflammatory adipokines (e.g., leptin, resistin), inducing lipotoxicity in trophoblast cells. This typically results in the placental release of copious exosomes enriched with pro-inflammatory mediators, exacerbating maternal insulin resistance [109]. Regular prenatal exercise not only controls fat mass but also significantly elevates the levels of adiponectin, a core adipokine with anti-inflammatory properties [118,133]. Moreover, exercise directly upregulates the placenta’s intrinsic capacity to synthesize adiponectin and Apelin [5,134]. This exercise-mediated optimization of the adipokine profile greatly enhances placental fatty acid oxidation capacity and mitochondrial biogenesis, protecting the placenta from lipotoxic damage. Because the cargo composition of placental exosomes is highly dependent on the redox state of the source cells (trophoblasts), the elimination of lipotoxicity ensures that the exosomal secretion mechanism remains undisturbed, thereby safeguarding the normal secretion of exosomes that promote physiological homeostasis [5,134].

In summary, prenatal exercise does not act on the placenta in isolation; rather, it systematically reshapes the trophoblast microenvironment through a multi-organ endocrine network involving skeletal muscle, adipose tissue, and the placenta. The exercise-induced increase in myokine secretion and the optimization of the adipokine profile synergistically reduce local oxidative stress and improve mitochondrial function, thereby indirectly maintaining the epigenetic homeostasis of trophoblast cells (such as DNA methylation and histone modification patterns) [5,135]. Under this ideal physiological and epigenetic homeostasis, the biogenesis mechanism of placental exosomes undergoes adaptive reprogramming. The vesicles released into the bloodstream become enriched with more protective bioactive substances that promote maternal–fetal angiogenesis, immune tolerance, and metabolic balance. This mechanism profoundly elucidates how exercise promotes the long-term health of both mother and infant through vesicular communication.

Beyond their established role as intercellular messengers, placenta-derived exosomes and exercise-induced humoral factors have recently garnered attention as accessible reservoirs of non-invasive biomarkers for real-time assessment of the maternal–fetal milieu. Concrete candidate biomarkers include: circulating exosomal microRNAs (e.g., *miR-505-5p*, *miR-351-5p*, and placenta-specific *miR-518b*), which reflect trophoblast stress, inflammatory status, and metabolic state; plasma exerkines such as apelin and irisin, whose gestational trajectories are directly modulated by maternal physical activity and correlate with placental angiogenesis, mitochondrial biogenesis, and fetal oxidative capacity; placental superoxide dismutase 3 (SOD3) activity and protein levels, which are detectable in maternal circulation and serve as a real-time readout of placental antioxidative reserve; cord blood DNA methylation marks at key metabolic and vascular genes (e.g., *CACNA1C*, *Nox4*, *Ppargc1a*), which provide a retrospective record of intrauterine epigenetic programming and offspring vascular predisposition; and maternal serum short-chain fatty acid (SCFA) concentrations, particularly acetate and butyrate, reflecting gut microbiota-derived signals that influence fetal neurodevelopment and metabolic patterning.

## 5. Direct Regulatory Mechanisms of Maternal Exercise on Embryonic Development

### 5.1. Regulation of Embryonic Gene Expression by Maternal Exercise via Epigenetics

Recent studies postulate that fetal developmental plasticity in utero allows the embryo to adjust its phenotype in response to environmental signals; if these adaptive alterations mismatch the postnatal environment, they may precipitate an increased risk of adult-onset diseases [22]. Traditional prenatal interventions have largely focused on nutritional management; however, emerging evidence demonstrates that maternal exercise of appropriate intensity not only benefits maternal health but also proactively reprograms the gene expression networks of the fetus [22,136]. Distinct from altering the DNA sequence, exercise exerts precise molecular regulation on the embryo via epigenetic mechanisms—modulating transcriptional activity by modifying chromatin structure without changing the underlying genetic code—thereby conferring long-lasting health advantages to the offspring.

**Figure 7:**
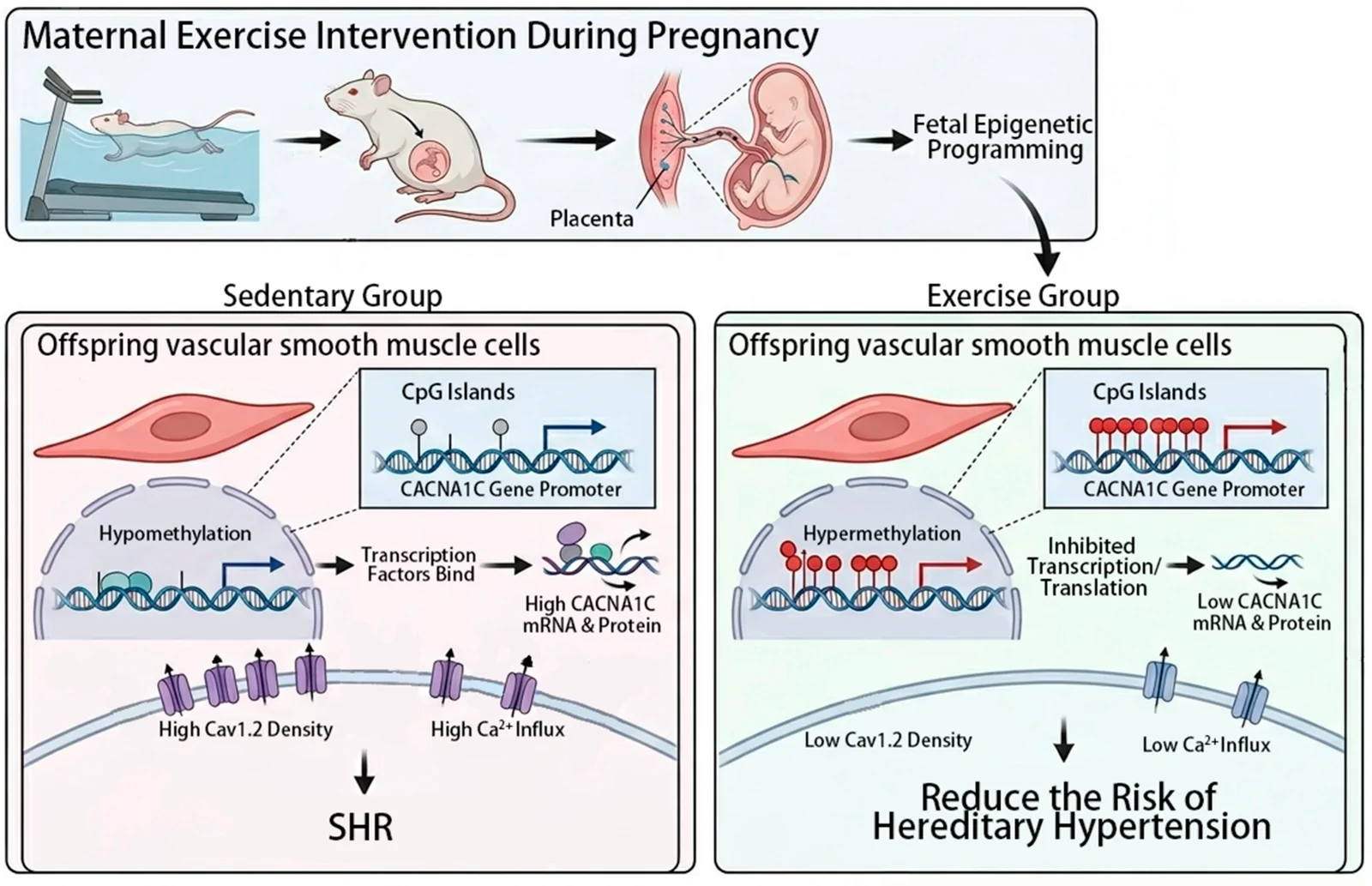
This figure depicts the transgenerational epigenetic mechanisms by which maternal exercise intervention during pregnancy mitigates the risk of hereditary hypertension in offspring. It illustrates how maternal exercise initiates fetal epigenetic programming in offspring vascular smooth muscle cells. In the sedentary group (**bottom left**), hypomethylation of CpG islands at the *CACNA1C* gene promoter permits transcription factor binding, resulting in high *CACNA1C* mRNA and protein expression; this upregulates Cav1.2 channel density and enhances calcium (Ca^2+^) influx, contributing to the development of hypertension (SHR). Conversely, in the exercise group (**bottom right**), exercise-induced hypermethylation of the *CACNA1C* promoter inhibits its transcription and translation, leading to lowered Cav1.2 density and reduced Ca^2+^ influx, highlighting the protective role of maternal lifestyle interventions in silencing hypertension-susceptibility genes via transgenerational epigenetic remodeling. Arrows indicate the sequence from maternal exercise to fetal epigenetic programming and vascular outcomes; blue and red CpG markers denote hypo- and hypermethylation, respectively.

DNA methylation is currently the most extensively studied epigenetic modification, typically occurring at CpG islands within gene promoter regions. Hypermethylation generally leads to transcriptional silencing, whereas hypomethylation promotes gene expression. Maternal exercise has been proven to precisely regulate the methylation levels of key developmental genes in the offspring [22]. In the spontaneously hypertensive rat (SHR) model, swimming exercise during pregnancy was found to significantly lower blood pressure in adult offspring. In-depth molecular investigations revealed that this long-term protective effect is rooted in the functional remodeling of L-type voltage-gated calcium channels (Cav1.2) in vascular smooth muscle cells. Specifically, maternal exercise induced significant hypermethylation in the promoter region of the *CACNA1C* gene (spontaneously hypertensive rat model) (which encodes the α1C core subunit of the Cav1.2 channel) in the mesenteric arteries of the offspring [137]. This epigenetic modification directly impedes the binding of transcription factors, thereby persistently suppressing the mRNA transcription and protein translation of *CACNA1C*. The ultimate physiological outcome is a reduction in calcium channel density in offspring vascular smooth muscle, decreased intracellular calcium influx, and consequently, a lowering of basal vascular tone and an enhancement of vasodilation. This discovery explains, for the first time, at the molecular level, how prenatal exercise sustains offspring vasodilation via epigenetic imprinting to counteract the risk of hereditary hypertension. Beyond specific genes, maternal exercise can also trigger genome-wide methylation rearrangements. Clinical analyses of umbilical cord blood samples post-exercise intervention revealed significant differences in genome-wide DNA methylation patterns between the exercise and control groups (human umbilical cord blood RCT). The study identified multiple differentially methylated regions (DMRs) involving key genes associated with metabolic regulation, immune responses, and cell signal transduction, such as *GPATCH2* [138]. This indicates that physiological signals generated by maternal exercise can cross the placental barrier or alter placental function to systematically reshape the methylation profile of fetal hematopoietic stem cells and other tissues. Furthermore, related reviews suggest that exercise may activate the AMPK/TET signaling axis in the fetal liver. TET enzymes are responsible for active DNA demethylation, implying that exercise can both intensify methylation and promote the expression of certain beneficial genes by activating demethylases, thereby forming a dynamic regulatory balance [22] (Figure 7).

**Figure 8:**
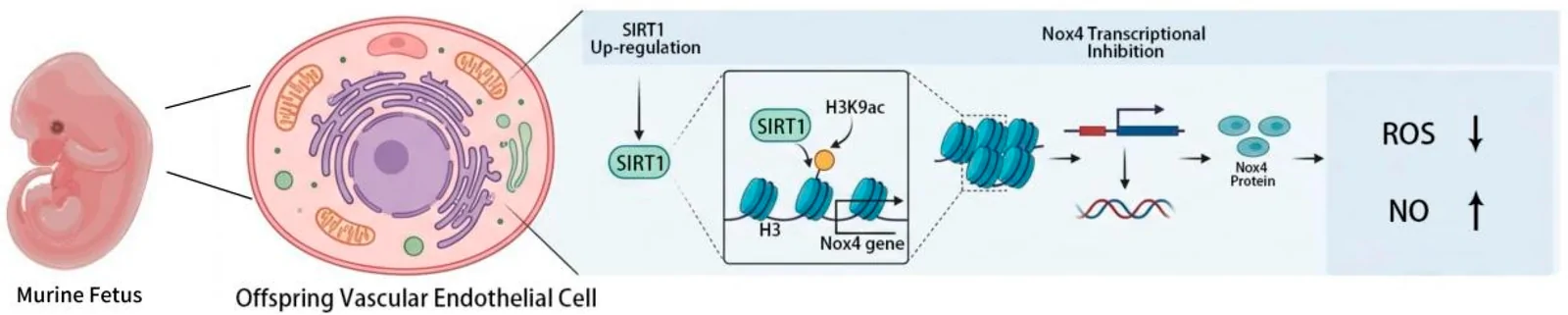
This figure depicts the epigenetic mechanism by which *SIRT1* up-regulation modulates oxidative stress in offspring vascular endothelial cells. It illustrates that up-regulated *SIRT1* targets histone H3 acetylation (H3K9ac) at the promoter region of the *Nox4* gene. This targeted epigenetic modification induces chromatin remodeling that leads to the transcriptional inhibition of the *Nox4* gene, thereby decreasing *Nox4* protein expression. Consequently, this targeted downregulation reduces the cellular levels of reactive oxygen species (ROS) while concomitantly increasing nitric oxide (NO) bioavailability, highlighting the crucial role of the *SIRT1*/*Nox4* signaling axis in mitigating endothelial oxidative damage and preserving vascular homeostasis. Arrows indicate the sequence from SIRT1 up-regulation to Nox4 transcriptional inhibition and the resulting decrease in ROS and increase in NO; colors distinguish molecular components.

Unlike DNA methylation, histone modifications finely tune transcriptional efficiency primarily by altering chromatin structure. In improving offspring vascular endothelial function, maternal exercise predominantly relies on antioxidant mechanisms mediated by histone modifications. Oxidative stress is the core pathological basis for vascular endothelial dysfunction and hypertension [139]. Research has shown that prenatal exercise significantly upregulates *SIRT1* expression in the vascular tissues of hypertensive offspring models (rat model of maternal hypertension). The increase in *SIRT1* directly targets a key enzyme of oxidative stress, NADPH oxidase 4 (*Nox4*). *SIRT1* deacetylates lysine 9 of histone H3 (H3K9ac) at the *Nox4* promoter region, leading to chromatin condensation in this area and thereby repressing *Nox4* transcription [48]. The downregulation of *Nox4* directly diminishes the generation of reactive oxygen species (ROS) within the vascular wall, restores endothelial nitric oxide synthase (eNOS) activity, and increases the bioavailability of the vasodilator NO. This mechanism demonstrates that prenatal exercise establishes a long-lasting antioxidant barrier for the offspring’s vascular system via histone deacetylation (Figure 8). Moreover, maternal exercise-induced alterations in placental gene expression exhibit almost-complete sexual dimorphism in male and female offspring. Through mouse models, exercise primarily led to the downregulation of genes related to lipid metabolism and steroid hormone synthesis (e.g., *Cyp11a1*) in male placentas. This may imply that under exercise conditions, male placentas tend to adjust the transport of energy substrates to prevent overgrowth or metabolic disorders. Conversely, in female placentas, exercise significantly upregulated genes associated with angiogenesis, muscle development, and extracellular matrix remodeling. The mechanistic basis for these sex-divergent transcriptomic responses lies in the interplay between sex-specific DNA methylation patterns and hormonal milieu [140]. In male placentas, exercise-induced downregulation of steroidogenic genes (e.g., *Cyp11a1*, *Hsd3b1*) may reflect a compensatory response to prevent excessive androgen exposure, which could otherwise disrupt fetal metabolic programming [119,141]. Conversely, female placentas upregulate angiogenic genes (e.g., *Vegfa*, *Angpt2*) and ECM remodelers (e.g., *Mmp2*, *Timp3*) in response to exercise, likely mediated by estrogen receptor-α (ERα) activation, which promotes endothelial proliferation and vascular stabilization [119,125,126,142]. Additionally, sex-dependent histone modification patterns—particularly H3K27ac and H3K4me3 enrichment at promoter regions of exercise-responsive genes—have been observed in mouse placentas, with females exhibiting broader chromatin accessibility at angiogenic loci [140,143]. These epigenetic differences are further reinforced by differential expression of epigenetic modifier enzymes (e.g., TET demethylases, histone acetyltransferases) between male and female trophoblasts, which may explain the sustained sex-specific programming effects even after the exercise stimulus ceases [144]. This suggests that female placentas may prioritize optimizing their vascular network structure to enhance nutrient uptake efficiency, thereby supporting fetal development [144]. Such sexual dimorphism may reflect an evolutionary adaptive strategy. Typically, male fetuses grow faster and are more sensitive to adverse intrauterine environments, while female fetuses possess greater survival resilience. Prenatal exercise acts as a physiological stimulus that drives the placenta to adopt distinct programming strategies based on fetal sex: emphasizing the maintenance of metabolic homeostasis for males, and structural/functional optimization for females. Additionally, studies indicate that exercise interventions can effectively mitigate the negative impacts of prenatal stress on the offspring’s brain HPA axis and glucocorticoid receptors (GR) [144], further substantiating the protective role of exercise on the neurodevelopment of offspring across sexes. The initiation of these epigenetic alterations stems from exercise stimulating maternal skeletal muscle, adipose tissue, and liver to secrete various bioactive molecules, such as Apelin, Irisin, and SERPINA3C [144]. These factors exert their effects primarily through two pathways: direct transplacental transmission, where small molecular factors cross the placental barrier into the fetal circulation to activate signaling pathways like AMPK and induce changes in epigenetic modifier activity; and placenta-mediated transmission, where exerkines bind to placental receptors, altering its nutrient transport and hormone secretion functions to indirectly optimize the fetal developmental environment.

Beyond these rodent-based mechanistic insights, emerging human cohort studies have substantiated that maternal exercise during pregnancy induces measurable DNA methylation alterations in offspring cord blood, providing direct translational evidence for exercise-mediated epigenetic programming in humans. In a randomized controlled trial of overweight/obese pregnant women, Yan et al. demonstrated that a structured cycling exercise program induced genome-wide differential DNA methylation in both maternal peripheral blood and umbilical cord blood. Notably, the promoter regions of genes involved in metabolic regulation, immune responses, and cell signal transduction exhibited significant methylation alterations, including CpG sites mapping to *GPATCH2* and other key regulatory loci. A systematic review of seven randomized controlled trials encompassing 1765 mother–child pairs further confirmed that lifestyle interventions combining diet and physical activity during pregnancy were consistently associated with genome-wide or gene-specific differential methylation and miRNA expression in cord blood or placental tissues, with the affected regions involving genes related to adiposity, metabolic processes, type 2 diabetes, birth weight, and fetal growth. Additional prospective cohort evidence from the NorthPop study supports the hypothesis that neonatal health effects related to maternal lifestyle may be partly mediated by epigenetic alterations in cord blood, with variably methylated loci identified as potential mediators linking prenatal exposures to offspring metabolic outcomes. These human data, while still limited in sample size and heterogeneous in intervention protocols, collectively corroborate the rodent findings and establish that maternal exercise can dynamically alter the offspring epigenome during gestation, with differentially methylated regions enriched in pathways relevant to long-term metabolic and cardiovascular health. An important conceptual consideration emerging from both human and animal studies is the partial reversibility of exercise-mediated epigenetic imprints. While certain DNA methylation and histone modifications induced by moderate maternal exercise exhibit remarkable persistence across childhood and even into subsequent generations—exemplified by the transgenerational demethylation of the Slc23a2 gene in offspring skeletal muscle—not all epigenetic marks are equally stable. The persistence of an epigenetic mark depends on multiple factors, including the specific genomic locus, the developmental window during which the modification was established, and the presence of ongoing environmental stimuli that either reinforce or erode the mark. Mechanistically, epigenetic modifications are maintained by DNA methyltransferases and TET demethylases; this balance remains responsive to postnatal environmental cues. Consequently, certain metabolic-related epigenetic modifications—particularly those affecting genes involved in energy homeostasis, insulin sensitivity, and adipocyte differentiation—may be partially offset by adverse postnatal lifestyle factors, including high-fat diets, sedentary behavior, or chronic stress. For example, the protective DNA methylation changes at the *Ppargc1a*/*PGC-1α* promoter induced by maternal exercise can be eroded by postnatal high-fat feeding, diminishing the metabolic benefits originally conferred by prenatal programming. Similarly, while maternal exercise-induced hypermethylation of the *CACNA1C* promoter in vascular smooth muscle cells provides sustained protection against hypertension, this epigenetic brake may be susceptible to attenuation under conditions of sustained oxidative stress or chronic inflammation in postnatal life. This nuanced understanding carries important clinical implications: maternal exercise should be viewed not as a one-time “epigenetic vaccination” that confers permanent protection, but rather as an early-life foundation that establishes a favorable baseline epigenetic landscape, which must be actively maintained through continued healthy lifestyle choices across the lifespan. Therefore, the optimal strategy for maximizing the long-term health benefits of prenatal exercise programming likely requires a life-course approach—integrating maternal exercise during pregnancy with sustained healthy dietary and physical activity patterns in the offspring from infancy through adulthood, thereby reinforcing and preserving the beneficial epigenetic imprints established in utero.

In summary, it is highly feasible that maternal exercise, acting as a potent environmental signal, regulates embryonic gene expression via epigenetics, though the specific molecular mechanisms remain an active area of future research. The epigenetic reprogramming data presented here—including *CACNA1C* methylation, *SIRT1*-mediated histone deacetylation, and genome-wide DMRs—originate almost exclusively from rodent studies. Notably, the human placental epigenome exhibits different CpG island distribution, hydroxymethylation dynamics, and transgenerational stability compared with mice. These interspecies differences necessitate cautious interpretation and underscore the urgent need for human cord blood and placental epigenetic studies with longitudinal follow-up.

### 5.2. Regulation of Embryonic Cellular Signaling Pathways by Maternal Exercise

Traditional obstetrical perspectives primarily focus on the role of prenatal exercise in controlling maternal weight and preventing gestational diabetes. However, cutting-edge molecular biological evidence demonstrates that physiological and biochemical changes produced during maternal exercise can cross the placental barrier and directly regulate developing embryonic cells. Maternal exercise during pregnancy can modulate multiple embryonic and placental cellular signaling pathways, with accumulating animal models and emerging human data indicating that these alterations confer long-term benefits on offspring growth, metabolism, and neurodevelopment [5,22].

Skeletal muscle is not only an organ of locomotion but also the largest metabolic organ in the human body. Research reveals that exercise can induce the maternal secretion of the exerkine Apelin. Apelin can cross the placenta, enter the fetal circulation, and bind to its receptor APJ. This binding triggers a cascade reaction in fetal muscle fibers, centered on the activation of the AMPK (AMP-activated protein kinase) signaling pathway [145,146]. During the embryonic stage, AMPK activation significantly promotes *PGC-1α* expression, driving mitochondrial biogenesis in fetal skeletal muscle. Furthermore, this pathway induces the expression of *UCP3* (uncoupling protein 3) and Sarcolipin, advancing the thermogenic gene program in skeletal muscle [145]. Results show that offspring mice subjected to prenatal exercise stimuli possess a higher proportion of oxidative muscle fibers in their skeletal muscles and a stronger capacity for fat-burning thermogenesis. Conversely, a lack of prenatal exercise leads to insufficient Apelin secretion and diminished AMPK pathway activity, subsequently triggering muscle metabolic dysfunction in the offspring [146]. Traditional hormones also play a vital role in the regulation of the fetus by prenatal exercise. The relationship between maternal exercise and fetal/neonatal thyroid hormone (T3) levels requires careful evidence stratification by species. In rodent gestation models, maternal exercise has been demonstrated to elevate embryonic and fetal T3 levels, which in turn activate thyroid hormone receptor (THR)-mediated transcription of myogenic regulatory factors (*MyoD*, *MyoG*) and promote skeletal muscle development. However, direct evidence linking maternal exercise to circulating T3 levels in human newborns remains limited. Existing human data are primarily derived from observational cohort studies reporting moderate correlations between maternal physical activity levels and cord blood thyroid hormone concentrations. For instance, cord serum T3 and T4 levels are typically lower than corresponding maternal levels across all gestational age groups, and the placental transfer of T3 from mother to fetus has been documented. Nevertheless, no randomized controlled human trial has directly tested whether prenatal exercise independently alters neonatal cord blood T3 concentrations after controlling for maternal thyroid status, gestational age, and other confounders. This evidence gap represents an important limitation: while the rodent data provide strong mechanistic plausibility, the translational relevance to human pregnancies—particularly regarding the magnitude and functional significance of exercise-induced T3 changes in the fetus—remains to be established through adequately powered human studies with direct fetal/neonatal thyroid hormone measurements. Upon entering embryonic muscle cells, T3 binds to the nuclear receptor THR (thyroid hormone receptor), directly regulating the expression of Myogenic Regulatory Factors (MRFs). The enhancement of this signaling pathway accelerates the proliferation and differentiation of embryonic myoblasts, ensuring healthy muscle mass and function at birth [84].

Neurodevelopment is exquisitely sensitive to the intrauterine environment. Multiple studies demonstrate that prenatal exercise can activate specific neuroprotective pathways, counteracting the negative impacts of genetic defects (e.g., autism risk) or environmental pressures (e.g., prenatal stress). In a valproic acid (VPA)-induced autism spectrum disorder (ASD) model, mechanistic studies via maternal swimming showed that VPA typically inhibits the Wnt/β-Catenin signaling pathway in the fetal brain, leading to hindered neurogenesis and increased apoptosis. However, exercise exerted a reversing effect on this inhibition (valproic acid-induced autism mouse model). Exercise stimulation increased Wnt ligands in the fetal hippocampus, promoting the accumulation of β-Catenin in the cytoplasm and its translocation to the nucleus, thereby initiating the transcription of downstream target genes. This process significantly enhanced the proliferative capacity of neural stem cells while suppressing the expression of pro-apoptotic proteins (such as *Bax*). Behavioral tests confirmed that this molecular-level repair directly translates to improved memory in the offspring [147]. Prenatal maternal stress typically leads to offspring HPA axis dysfunction and cognitive deficits. The literature indicates that prenatal exercise exerts a protective role by upregulating *BDNF* (brain-derived neurotrophic factor) and VEGF (vascular endothelial growth factor) in the hippocampus [148]. Exercise not only restores the stress-induced downregulation of *BDNF*/VEGF expression but also normalizes the offspring’s HPA axis reactivity by modulating glucocorticoid receptor (GR) sensitivity. This implies that prenatal exercise actively builds stress resilience in the fetal brain, making it more adaptable to future environmental pressures. Moreover, maternal brain plasticity also plays a role in this process, potentially influencing the fetus indirectly via neuroendocrine pathways [149].

In the placenta, exercise can also activate metabolic pathways, thereby improving trophoblast differentiation, angiogenesis, lipid metabolism, and epigenetic programming. Similar to skeletal muscle, placental *PGC-1α* is also activated by maternal exercise. The upregulation of *PGC-1α* further promotes the expression of *FNDC5*, which is cleaved into the secreted protein Irisin [5,150]. Irisin plays a crucial pro-angiogenic role in the placenta, particularly within the Labyrinth Layer. Studies indicate that Irisin enhances the density and branching complexity of the placental vascular network. Additionally, exercise can differentially regulate the expression of placental growth factors VEGF and PlGF, as well as their receptors, preventing fetal growth restriction caused by insufficient angiogenesis [125]. In the pathological state of fetal growth restriction (FGR), placental mTOR signaling is often suppressed, leading to abnormal lipid metabolism. Current literature suggests that moderate prenatal exercise may help maintain the normal activity of placental mTOR signaling (mouse model of fetal growth restriction), thereby improving nutrient transport [151]. Notably, placental response to exercise exhibits sexual dimorphism; male and female placentas display divergent adaptive strategies in gene expression in response to maternal exercise [119].

In summary, these studies indicate that maternal exercise, acting as a systemic stimulus, coordinates signal transduction changes along the maternal-placental-fetal axis via exerkines and placental factors, thereby regulating the physiological functions of the offspring. However, almost all mechanistic studies have been conducted in rodents; the specific regulatory mechanisms of core embryonic patterning pathways (canonical Wnt, BMP, Notch) in normal embryos remain poorly understood and can only be inferred from disease models (e.g., autism, stress) rather than physiological pregnancies. Most of the signaling pathway modulation (Apelin–AMPK, thyroid hormone–MRF, Wnt/β-catenin, and *BDNF*/VEGF) has been characterized in mouse and rat models. While these pathways are evolutionarily conserved, the dose–response relationships, receptor affinities, and cross-talk with human gestational hormones (e.g., hCG, estriol) remain poorly understood. Hence, direct clinical translation of these rodent-based signaling mechanisms should be made with explicit acknowledgment of species-specific differences.

### 5.3. Regulation of Embryonic Oxidative Stress and Apoptosis Balance by Maternal Exercise

During mammalian embryonic development, Reactive Oxygen Species (ROS) play a pivotal regulatory role. Physiological concentrations of ROS serve as essential signaling molecules for angiogenesis, cell signal transduction, and differentiation; however, when the mother is exposed to pathological environments such as obesity, diabetes, hypertension, or hypoxia, excessive ROS can attack cellular lipids, proteins, and DNA, leading to mitochondrial dysfunction, which in turn activates the Caspase cascade and triggers pathological apoptosis [152]. The placenta, as the core hub at the maternal–fetal interface, has a functional integrity that directly determines the fate of the embryo. Recent research has focused on how prenatal exercise, acting as a systemic physiological stimulus, maintains normal embryonic development under oxidative stress by remodeling the placental antioxidant defense system and blocking deleterious apoptotic signals at the molecular level [126].

Maternal exercise reverses stress-related placental dysfunction primarily by releasing multiple bioactive factors. First, Apelin plays a central role in combating obesity-related placental dysfunction. Exercise significantly elevates maternal circulating Apelin levels (mouse model of maternal obesity), which, upon acting on the placenta, upregulates the expression of PR domain containing 16 (*PRDM16*) [153]. *PRDM16*, a key regulator of mitochondrial biogenesis, drives the shift in placental cellular energy metabolism from glycolysis to oxidative phosphorylation. This metabolic remodeling not only replenishes the placental energy reserves to combat oxidative stress but also optimizes nutrient transport to the fetus. Second, exercise-induced Irisin exhibits direct anti-apoptotic effects (human placental explant study). Under oxidative stress conditions, Irisin binds to integrin receptors on the placental surface to activate the downstream Akt signaling pathway. Phosphorylated Akt, on one hand, promotes the expression of antioxidant enzymes, and on the other hand, directly inhibits the translocation of the pro-apoptotic protein Bax to the mitochondrial membrane, preventing the formation of transmembrane pores in the outer mitochondrial membrane. This sustains the mitochondrial membrane potential and curtails the apoptotic program at its source [154]. Additionally, in healthy pregnancies, exercise significantly increases the expression of placental Angiogenin (human placental analysis), promoting the expansion of the villous vascular network without triggering endoplasmic reticulum stress (ER Stress) [142]. This indicates that moderate exercise creates a beneficial microenvironment that effectively maintains intracellular redox homeostasis while enhancing placental functional reserves.

Faced with pathological stressors such as maternal hypertension, diabetes, or perinatal hypoxia, prenatal exercise demonstrates a significant preconditioning protective effect. In offspring subjected to hypoxic–ischemic stress, those in the exercise group exhibited significantly lower expressions of the pro-apoptotic proteins Bax and *Caspase-3* (neonatal hypoxia-ischemia rat model) in the hippocampus, whereas the levels of the anti-apoptotic protein Bcl-2 and its upstream neurotrophic factor *BDNF* were significantly elevated. This positive remodeling of the Bcl-2/Bax ratio effectively reduced neuronal loss and improved the cognitive and memory functions of the offspring [155]. In diabetic and obese models, exercise has been proven to comprehensively restore the activities of SOD1, catalase (*CAT*), and glutathione peroxidase (GPx). The comprehensive activation of this antioxidant enzyme system effectively neutralizes lipid peroxidation (LPO) induced by high-glucose and high-fat environments, preserving the integrity of embryonic cell membranes [120,156].

Notably, recent research has revealed that the protective effects of exercise on the maternal–fetal interface possess significant sex specificity [119]. Transcriptomic analysis demonstrates marked differences in the responses of male and female placentas to maternal exercise (mouse study). The sex-specific oxidative stress responses observed in exercise-exposed placentas may arise from differential basal antioxidant capacity between male and female tissues. Female placentas generally exhibit higher expression of antioxidant enzymes (SOD1, GPx4, catalase) and lower baseline reactive oxygen species (ROS) levels, which may render them more responsive to exercise-induced upregulation of *Nrf2*-dependent antioxidant genes. In contrast, male placentas, with inherently higher metabolic activity and ROS production, may prioritize mitochondrial quality control pathways (e.g., *PGC-1α*, mitophagy) rather than direct ROS scavenging. Additionally, sex hormone signaling modulates apoptotic thresholds: estrogen enhances Bcl-2 expression and suppresses Bax translocation via PI3K/Akt, while testosterone may sensitize cells to pro-apoptotic stimuli through MAPK pathway activation. This hormonal influence, combined with sex-biased exerkine receptor distribution (e.g., higher APJ expression in female placental endothelial cells), may explain the more robust anti-apoptotic phenotype observed in female placentas following maternal exercise. For example, male placentas exhibit a more pronounced anti-inflammatory and mitochondrial gene response following exercise stimulation. This suggests that male and female embryos may derive distinct specific metabolic benefits from maternal exercise. This discovery not only highlights the complexity of oxidative stress regulation but also provides novel insights for the future development of personalized, precision prenatal exercise prescriptions.

In summary, moderate prenatal exercise can effectively regulate the dynamic balance between oxidation and antioxidation in the placenta and embryonic tissues. This benefit is achieved primarily by upregulating antioxidant defense mechanisms, improving mitochondrial metabolism, and intervening in key apoptotic pathways. This not only robustly supports fetal intrauterine development but also lays a solid foundation for the long-term health of the offspring. However, the actual protective efficacy remains comprehensively influenced by the mother’s baseline health status, exercise load, and genetic background. The anti-apoptotic and antioxidant mechanisms (e.g., Apelin–*PRDM16*, Irisin–Akt, SOD/*CAT* upregulation) have been validated mainly in rodent models of hypertensive, diabetic, or hypoxic pregnancies. However, the human placenta has a much more complex villous structure, greater metabolic capacity, and distinct reactive oxygen species buffering systems. Moreover, the sex-specific responses observed in mouse placentas have not been systematically replicated in human cohorts, limiting the translational value of current sex-dimorphic findings.

## 6. Discussion

### 6.1. Practical Prenatal Exercise Recommendations Based on Current Evidence

Beyond the molecular and mechanistic insights discussed above, translating these findings into actionable prenatal care requires clear, evidence-based exercise prescriptions that are both safe and effective. Current guidelines from the American College of Obstetricians and Gynecologists (ACOG) recommend that pregnant women without contraindications accumulate at least 150 min of moderate-intensity aerobic activity per week throughout pregnancy [157]. This recommendation is supported by robust evidence showing reduced risks of gestational diabetes, hypertensive disorders, and excessive gestational weight gain, without increasing the risk of preterm birth or low birth weight [158].

Based on the synthesis of available human cohort and interventional studies, the following modalities have been most extensively validated and are considered safe for healthy pregnancies: brisk walking—the most commonly studied and accessible form, with proven benefits for glycemic control and maternal fitness; moderate swimming or water aerobics—offering buoyancy-mediated joint relief and reduced injury risk, with favorable effects on maternal hemodynamics; stationary cycling—allowing precise intensity monitoring and low fall risk; and low-resistance strength training—focusing on major muscle groups with light weights or elastic bands, which improves musculoskeletal conditioning without inducing excessive maternal cardiac strain.

Session duration typically ranges from 20 to 45 min per session, performed 3–5 times per week, with gradual progression from lower to higher volumes in the first trimester. Absolute contraindications include hemodynamically significant heart disease, restrictive lung disease, incompetent cervix, placenta previa after 26 weeks, premature labor, ruptured membranes, and preeclampsia with severe features. Relative contraindications, such as anemia, chronic bronchitis, or poorly controlled type 1 diabetes, require individualized medical clearance and close supervision.

Importantly, the evidence base for these recommendations is predominantly derived from healthy, normal-weight pregnant women in high-income settings. For high-risk subgroups—including those with pre-existing obesity (BMI ≥ 30 kg/m^2^), gestational diabetes on insulin therapy, chronic hypertension, multiple gestations, or a history of preterm delivery—the optimal exercise dose, type, and timing remain poorly defined. The few available studies in these populations are underpowered and often confounded by concomitant dietary or pharmacological interventions. Consequently, current ACOG guidelines necessarily rely on expert consensus rather than high-grade evidence for these subgroups. This knowledge gap is particularly concerning given that these very populations stand to gain the most from lifestyle modifications. Future pragmatic randomized trials are urgently needed to establish trimester-specific, phenotype-tailored exercise prescriptions that maximize fetal developmental benefits while minimizing maternal and neonatal risks, thereby closing the loop between mechanistic DOHaD research and equitable clinical implementation. The exercise modalities and related evidence are summarized in Table 1.

### 6.2. Cross-Study and Cross-Species Limitations: Exercise Modality, Metabolic Adaptation and Evidence Heterogeneity

Several important methodological and biological limitations must be acknowledged when interpreting the evidence synthesized in this review. First, substantial discrepancies exist between rodent exercise paradigms and human prenatal physical activity prescriptions. Most animal studies cited herein employed either voluntary wheel running (mice) or forced swimming/treadmill training (rats), with exercise volumes, intensities and durations that are difficult to standardize or translate to human settings. Typical rodent protocols involve ad libitum wheel access (often 2–6 km/night) or daily 20–60 min swimming/treadmill sessions, whereas human prenatal guidelines recommend structured moderate-intensity aerobic exercise or low-resistance strength training at 150 min/week. The gestational intervention window also differs fundamentally: rodents are typically exercised throughout gestation (≈19–21 days), which covers late organogenesis and rapid fetal growth, whereas human interventions often begin in the second trimester (weeks 12–20) and continue to term, thereby missing the first-trimester organogenetic peak. Furthermore, spontaneous physical activity patterns (circadian rhythms, nesting behavior, home-cage ambulation) in sedentary control rodents introduce baseline activity variability that is absent in human sedentary controls. These interspecies and inter-protocol differences limit the direct quantitative extrapolation of dose–response relationships from animal models to clinical prenatal exercise prescription.

Second, the interpretation of exercise-induced metabolic effects requires a clear distinction between chronic exercise-mediated adaptive metabolic remodeling and static basal metabolic rate (BMR). Many human studies adjust outcomes for maternal BMR or total energy expenditure, but this approach does not capture the sustained epigenetic and hormonal reprogramming induced by regular physical activity—effects that persist independently of resting metabolism. Maternal exercise drives activity-dependent transcriptional changes (e.g., AMPK-*PGC-1α* signaling, myokine secretion, DNA methylation remodeling) that operate through pathways distinct from the simple caloric deficit or BMR elevation typically associated with increased energy expenditure. Thus, the metabolic benefits observed in offspring are not merely a consequence of reduced gestational weight gain or improved maternal BMR, but rather reflect exercise-specific molecular adaptations that continue to influence placental function and fetal gene expression even when BMR is statistically accounted for. The failure to differentiate these two levels of regulation has led to over-simplified interpretations in some epidemiological studies, where exercise effects are partially or incorrectly attributed to baseline metabolic differences.

Third, the overall evidence base suffers from pronounced heterogeneity across multiple dimensions: (1) most mechanistic insights are derived from single-animal-model studies (often one rodent strain per publication), with limited cross-strain or cross-species validation; (2) human cohort sample sizes are frequently small (many placental analyses *n* < 50) and underpowered for sex-stratified or subgroup analyses; (3) intervention protocols vary widely in frequency, intensity, type and gestational timing, precluding direct meta-analysis of combined outcomes; and (4) confounding factors—including pre-pregnancy BMI, age, parity, dietary background, socioeconomic status and baseline fitness—are inconsistently controlled, even in well-designed cohort studies. This heterogeneity complicates the identification of robust effect sizes and limits the generalizability of current conclusions to diverse maternal populations. Large-scale, multicentre, prospectively registered RCTs with standardized exercise prescriptions and comprehensive confounder adjustment are urgently needed to strengthen the causal evidence base and to enable meaningful comparisons across studies.

Despite the mechanistic plausibility of sex-divergent placental and fetal responses to maternal exercise, human sex-stratified data remain critically insufficient. The overwhelming majority of mechanistic evidence for sex-specific placental adaptations—including transcriptomic, epigenetic and signaling differences—derives from mouse models, where genetic background, housing conditions and exercise protocols are tightly controlled. In human cohorts, most studies lack adequate statistical power for sex-stratified analyses, and even when sex is included as a covariate, sample sizes are rarely sufficient to detect interaction effects. Moreover, confounding by fetal sex-linked differences in growth trajectories, maternal hormonal profiles and placental sampling timing complicates interpretation. The translational relevance of rodent sex-specific findings to human pregnancy is further limited by differences in placental architecture (hemochorial in humans vs. haemotrichorial in mice), gestational length and sex chromosome dosage compensation mechanisms. Therefore, while the mechanistic framework presented here provides plausible hypotheses, clinical recommendations should not assume sex-specific exercise effects until large-scale, prospectively designed human studies with pre-specified sex stratification are completed and their findings replicated across diverse populations. Future research must prioritize adequately powered RCTs with mandatory sex-disaggregated reporting, coupled with longitudinal offspring follow-up, to determine whether the sex-divergent programming observed in animals is clinically meaningful in humans.

While the evidence for exercise-induced modulation of fetal adiposity is promising, several caveats merit attention. First, although meta-analytic data confirm that prenatal physical activity reduces macrosomia risk without altering mean birth weight, most human studies rely on indirect measures of neonatal adiposity (e.g., skinfold thickness, bioelectrical impedance) rather than gold-standard methods such as MRI or DXA, which limits precision in quantifying fat-specific changes. Second, the distinction between subcutaneous and visceral fat depots—which have divergent metabolic implications—is rarely reported, yet visceral adiposity carries stronger associations with later cardiometabolic risk. Third, the optimal window for exercise intervention to modulate fetal fat accretion remains unclear; most interventions begin in the second trimester, whereas the most rapid fat deposition occurs in the third trimester. Fourth, maternal nutritional status and pre-pregnancy BMI are powerful confounders that may modify or even reverse the exercise-fat relationship [92]. Finally, the physiological necessity of fetal fat—particularly brown adipose tissue for thermogenesis—means that the “optimal” fat level is not “as low as possible,” but rather a range that balances short-term neonatal survival with long-term metabolic health. Clinicians should interpret reported fat-reduction effects with this dual perspective, avoiding the simplistic narrative that all fetal fat reduction is inherently beneficial.

## 7. Conclusions

This review integrates preclinical and clinical evidence to outline the regulatory network underlying maternal exercise-mediated fetal developmental programming. Maternal exercise influences embryonic organogenesis and fetal development not through a single circulating factor or a direct mechanical effect on the fetus, but through coordinated remodeling of the maternal–placental–fetal environment. Exercise-induced improvements in maternal metabolic, immune and redox homeostasis are transmitted across the placental interface through altered perfusion and nutrient transport, exerkines and extracellular vesicles, microbiota-derived metabolites, and epigenetic and nutrient-sensing pathways. These convergent signals subsequently regulate organ-specific processes, including neurogenesis and synaptic maturation, cardiovascular and pulmonary development, intestinal epithelial and enteric nervous system maturation, and skeletal muscle and bone formation. Thus, the placenta functions as the principal integrative interface that determines whether maternal exercise signals are conveyed, modified or buffered before reaching the developing fetus.

Maternal nutrition is an essential component of this framework because it supplies the metabolic substrates and signaling molecules required for fetal growth and can modify both maternal exercise adaptations and placental responses. However, the available evidence does not yet establish that combined exercise and nutritional interventions consistently produce additive or synergistic developmental benefits. Human evidence is strongest for improvements in maternal metabolic health and placental structure and function, whereas most organ-specific, epigenetic and microbiota-mediated mechanisms remain derived from animal models. Therefore, the principal implication of this review is not that a universal diet–exercise combination can currently be prescribed, but that fetal developmental programming should be investigated as an integrated and context-dependent exercise–nutrition process. Future translation will require standardized, adequately powered human trials that directly compare exercise alone, nutrition alone and combined interventions, while accounting for gestational timing, exercise dose, maternal metabolic status, dietary background and fetal sex. Such evidence is necessary to transform the present mechanistic framework into precise and clinically applicable prenatal lifestyle strategies. The cross-system evidence is summarized in Table 2.

## Figures and Tables

**Table 1:** Summary of Evidence for Moderate Prenatal Exercise Modalities, Intensity and Clinical Outcomes. Arrows indicate increases (↑) and decreases (↓).

Modality	Recommended Frequency & Duration (per ACOG)	Key Reported Clinical Outcomes (Human Evidence)	Corresponding Mechanistic Evidence (Animal Models)	Evidence Gaps for High-Risk Populations
Brisk walking	≥150 min/week; 30–45 min/session, 3–5×/week; RPE 12–14	↓ GDM risk (~40% reduction), ↓ excessive GWG, ↓ fasting glucose, ↓ maternal CRP levels	Improved placental GLUT1 regulation (rodent); upregulated *SIRT1*/*Nox4* antioxidant axis (SHR model)	No dose–response data in obese (BMI ≥ 30) or GDM-insulin-treated women; optimal timing in 3rd trimester unclear
Moderate swimming/water aerobics	≥150 min/week; 20–40 min/session; RPE 12–14	Improved fetal cardiac outflow parameters; ↓ maternal joint pain; ↓ risk of macrosomia	Enhanced placental VEGF/PlGF expression and angiogenesis (mouse); ↑ placental FATP4 for fatty acid transport	Safety data lacking in cervical insufficiency or placenta previa; effects on fetal breathing movements under water not quantified
Stationary cycling	≥150 min/week; 20–45 min/session; RPE 12–14	↓ gestational hypertension risk; stable maternal hemodynamics; no adverse fetal heart rate changes	Activation of placental *PGC-1α*/*FNDC5*/Irisin pathway (mouse); ↑ mitochondrial biogenesis in trophoblasts	No RCTs in chronic hypertensive or twin pregnancies; intensity titration protocols not standardized
Low-resistance strength training	2–3×/week; 15–30 min/session; light weights/elastic bands; RPE 12–14	Improved lumbar-pelvic stability; ↓ low back pain; maintained lean mass; no increase in preterm contractions	Upregulation of myogenic regulatory factors (*MyoD*, *MyoG*) via thyroid hormone signaling (rat); mechanical YAP/FAK activation in musculoskeletal progenitors	Safety of Valsalva maneuvers and intra-abdominal pressure changes not assessed; effects on placental perfusion during resistance bouts unknown
General moderate-intensity aerobic activity (mixed/unspecified)	As above (meta-analyses)	↓ preeclampsia risk (pooled OR ~0.75); ↓ neonatal respiratory distress; improved offspring neurocognitive scores at 1–3 years	Gut–brain axis modulation via SCFAs and 5-HT (mouse); *BDNF* upregulation in fetal hippocampus	Sex-specific responses in humans unconfirmed; most human cohort data limited to self-report without objective accelerometry

**Table 2:** Summary of Core Evidence by Developmental System. Arrows indicate increases (↑) and decreases (↓).

Developmental System	Main Evidence Sources	Common Exercise Modalities	Key Fetal/Offspring Outcomes	Evidence Strength & Notes
Nervous system	Mouse/rat models (high-fat diet, autism, protein restriction); limited human cohorts	Voluntary wheel, treadmill, swimming	↑ *BDNF*, ↑ hippocampal neurogenesis, ↑ synaptic plasticity; gut–brain axis (SCFA, 5-HT, GABA)	Animal mechanism strong; human RCT/cohort limited (small *n*, self-report)
Cardiovascular system	Rat (SHR, GDM); human pilot ultrasound	Swimming, treadmill, supervised aerobic	↓ *Nox4*/ROS via *SIRT1*, ↑ NO, ↑ cardiac outflow, ↑ ventricular filling	Rodent epigenetic mechanism well-defined; human imaging data preliminary
Digestive system	Mouse/avian embryos; rodent stress models	Maternal exercise (indirect mechanical)	YAP/FAK mechanotransduction, RhoA/ROCK activation, relieved Wnt/Notch inhibition	Mostly embryonic animal models; human translation speculative
Respiratory system	Human birth cohort (*n* = 2366); rodent hypoxia models	Self-reported exercise (≥3×/week); various aerobic	↓ Childhood asthma risk, ↑ lung function at 3 months; improved placental perfusion	Strong human epidemiological data; mechanistic pathways inferred from animals
Musculoskeletal system	Mouse (obesity, akinesia); rat	Treadmill, voluntary wheel, swimming	↑ *MyoD*/*MyoG* via T3/TR, Apelin–AMPK–*PGC-1α*, ↓ *Ppargc1a* methylation, ↑ bone volume	Rodent models predominant; human data scarce
Maternal metabolism (GDM, insulin resistance)	Human RCT meta-analyses; human + rodent metabolomics	Moderate aerobic (≥150 min/week), low-to-moderate intensity	↓ GDM risk ~43%, ↓ BCAA, ↑ arginine/citrulline, ↓ leptin/TNF-α, ↑ IL-6 (myokine)	Strong human RCT/cohort evidence; mechanistic details from rodent
Maternal immune & oxidative balance	Human placental + rodent; human cohorts	Aerobic exercise	↓ NLRP3 inflammasome, ↓ IL-1β/IL-18, ↑ IL-1ra/IL-10, ↑ SOD/GPx, ↑ HDL function	Human and animal data convergent; some obesity-specific limitations
Placental structure & transport	Human cohorts (meta-analysis, *n* = 119); mouse sex-specific studies	Various exercise modalities	↑ Villous density, ↑ VEGF/VEGFR, ↑ FATP4, sex-specific gene expression (male ↓ lipid, female ↑ angiogenesis)	Human morphological data solid; sex-specific differences mostly from mice
Placental exosome & signaling	Mouse + human placental explant	Exercise-induced myokines (Apelin, Irisin)	Apelin/AMPK, Irisin/Akt, altered exosomal miRNA/protein cargo (anti-inflammatory, pro-angiogenic)	Animal + human ex vivo; in vivo human exosome data emerging
Epigenetic reprogramming	Rat (SHR) + human umbilical cord RCT (*n* = 24); mouse sex-specific	Swimming, cycling (RPE 12–14, 3×/week)	↑ *CACNA1C* methylation, genome-wide DMRs (*GPATCH2*), *SIRT1*/H3K9ac at *Nox4*, sex-divergent placental methylome	Rodent transgenerational evidence; human RCT small but supportive
Signaling pathways	Mouse/rat models (obesity, autism, stress, FGR)	Treadmill, swimming, voluntary	Apelin–AMPK–*PGC-1α*, T3–THR–MRF, Wnt/β-Catenin, *BDNF*/VEGF, placental mTOR	All mechanistic data from rodents; human translation requires caution
Oxidative stress & apoptosis	Mouse/rat (obesity, diabetes, hypoxia-ischemia); human placental explant	Treadmill, swimming	Apelin–*PRDM16*, Irisin–Akt, ↑ SOD/*CAT*/GPx, ↓ Bax/*Caspase-3*, ↑ Bcl-2, ↑ *BDNF*	Animal models dominate; human ex vivo confirms some pathways
Gut microbiota–metabolite axis	Mouse + human observational	Various exercise modalities	↑ α-diversity, ↑ SCFAs, ↑ tight junctions (ZO-1/Occludin), ↓ LPS/TLR4/NF-κB, HDAC inhibition via butyrate	Rodent mechanism + human correlational data; causal link not fully proven

## Data Availability

No new data were created or analyzed in this study. Data sharing is not applicable to this article.
